# Regional high-frequency monitoring revealed chloride concentrations in exceedance of ecological benchmarks in urban streams across the Delaware River Basin, USA

**DOI:** 10.1007/s10661-025-14485-6

**Published:** 2025-08-29

**Authors:** Rosemary M. Fanelli, Michelle Morency, Brandon J. Fleming, Joel Moore, Deanna Hardesty, Megan Shoda

**Affiliations:** 1 U.S. Geological Survey South Atlantic Water Science Center, Raleigh, NC USA; 2 U. S. Geological Survey Pennsylvania Water Science Center, New Cumberland, PA USA; 3 Towson University, Towson, MD USA; 4 U.S. Geological Survey Water Mission Area, Reston, VA USA

**Keywords:** Stream ecosystems, Freshwater salinization, Deicer applications, Urbanization, Water quality, Chloride, Delaware River Basin

## Abstract

Rising chloride concentrations pose critical risks to freshwater stream ecosystems in temperate regions like the Delaware River Basin (DRB), USA, where winter deicer applications (*i.e.*, road salt) are common. Increasing chloride concentrations have been documented in the region, but the extent to which chloride exceeds regulatory benchmarks remains unclear because detection of exceedances requires continuous monitoring of chloride (*i.e.*, hourly or daily). A network of 82 non-tidal continuous specific conductance (SC) monitoring sites, spanning varied land use and geological settings, was established across the DRB to address this research need. First, a cluster analysis was conducted to group sites based on their watershed characteristics. Next, regression models for sites and clusters were developed to predict chloride using SC as a proxy. Finally, daily mean and hourly mean chloride concentration predictions were made for a three-year period (2020–2022) at the 82 study sites and analyzed to determine where and when chloride exceeded federal regulatory benchmarks. Chloride exceedance events occurred at 35% of the sites, all of which had 5% impervious cover or greater. Seasonally elevated chloride also was predicted at sites with less than 5% impervious cover. Variability in chloride patterns likely was influenced by deicer material types, winter weather patterns, geological settings, and gaps in data coverage. This study demonstrated the value of SC as a proxy for predicting chloride concentrations and showed how SC-chloride regression relationships vary across settings. More broadly, this study highlighted the value of continuous water quality monitoring to assess effects of freshwater salinization at a regional scale.

**Supplementary Information:**

The online version contains supplementary material available at 10.1007/s10661-025-14485-6.

## Introduction

Increasing ion concentrations in freshwater streams and rivers are a globally recognized concern, with far-reaching impacts for aquatic ecological integrity and human health (Hintz & Relyea, 2019; Kaushal et al., 2021). A majority of studies on aquatic insects reported negative responses to increased salinity in a recent global meta-analysis (Walker et al., 2023); responses include reduced growth and development (Johnson et al., 2015), as well as increased drift and mortality (Clements & Kotalik, 2016). Elevated levels of chloride and sodium ions—which are naturally low in most freshwater systems—disrupt osmoregulatory processes in benthic macroinvertebrates (Griffith, 2017) and trigger changes to gill microstructure and altered protein expression (Orr et al., 2023). Shifts in dominant taxa or functional feeding groups at the community scale also have been associated with increased ion concentrations (Castillo et al., 2018; Hintz & Relyea, 2019). Drinking water supplies are at risk from elevated chloride that may lead to corrosion of drinking water infrastructure and release of trace metals, including lead and copper, into municipal drinking water systems (Stets et al., 2018). Greater mobilization of radionuclides in groundwater sources in response to increasing salinity also has been observed (Lazur et al., 2020; Lindsey et al., 2021).

Ecological benchmarks have been used to assess the degree to which freshwater salinization may be impairing the ecological integrity of freshwater ecosystems. For example, a national background conductivity dataset (Olson & Cormier, 2019) recently was used to assess departures from background conductivity in non-tidal streams across the Chesapeake Bay watershed in the mid-Atlantic USA (Fanelli et al., 2024). Aquatic toxicity levels differ between ions (Clements & Kotalik, 2016), however, and since conductivity reflects total ion concentration, the application of ion-specific benchmarks can be more useful for characterizing effects of rising salinity on aquatic ecosystems. The only current national major ion benchmark for aquatic life protection related to salinity is the U.S. Environmental Protection Agency’s (EPA) chronic and acute chloride criteria, which are 230 mg L^−1^ for a four-day rolling average or 860 mg L^−1^ for one hour, respectively (U.S. EPA, 1988). Multiple states, including Maryland (Maryland Department of the Environment, 2022), Virginia (Virginia Department of Environmental Quality, 2017) and New Hampshire (New Hampshire Department of Environmental Services, 2008) have used these benchmarks to document elevated chloride and/or list water bodies on their state 303(d) impairment list, which is a list each state provides to the EPA of water bodies that do not meet water quality standards. Canada has established more conservative chronic and acute chloride criteria for aquatic organisms (120 and 640 mg L^−1^, respectively; Canadian Council of Ministers of the Environment, 2011). These regulatory benchmarks have been applied to assess effects of urbanization and deicer applications on water quality in temperate regions, with results showing exceedances associated with greater impervious cover (Corsi et al., 2015; Moore et al., 2020; Weatherson et al., 2024).

High frequency (*i.e.,* continuous) water quality monitoring is necessary to accurately document exceedance events above regulatory benchmarks. This type of monitoring typically uses an in situ sensor that takes measurements at regular intervals (*e.g.,* every 60 min). Discrete water quality sampling, in contrast, is conducted more infrequently (*i.e.*, greater than 24 h between measurements), and does not capture dynamic in-stream chloride conditions needed to compare to regulatory benchmarks (Moore et al., 2020). Sensor networks for continuous water quality monitoring have been used to characterize dynamic conditions in streams and rivers for a variety of water quality parameters (Horsburgh et al., 2010; Rode et al., 2016). Although continuous monitoring is ideal for capturing dynamic conditions, reliable, ion-specific sensors are lacking for chloride, so regression models between chloride concentration and specific conductance (SC, electrical conductivity at 25 °C measured in microsiemens per centimeter, or µS cm^−1^) often are used for continuous chloride prediction. SC has successfully been used as a predictor of chloride (Rossi & Gyves, 2023), and the combination of discrete measures of chloride and continuous SC monitoring has proven an effective way to assess chloride exceedances above regulatory benchmarks (Moore et al., 2020; Weatherson et al., 2024).

The purpose of this study was to characterize the extent and severity of exceedances of chloride concentrations above established regulatory benchmarks in an ecologically diverse, temperate region impacted by freshwater salinization. A continuous SC sensor network was recently established in the Delaware River Basin (DRB) by the U.S. Geological Survey (USGS), in partnership with local and regional stakeholders, to better understand effects of freshwater salinization on water availability in the region (Eberts et al., 2019). Previous research in the DRB highlighted increasing trends in multiple indicators of salinity, including SC, total dissolved solids, chloride, and sodium (Shoda & Murphy, 2022). A follow up study examining trends in SC and streamflow showed seasonal increases in SC during winter seasons in urban areas, suggesting deicer applications as a driver of these trends (Rumsey et al., 2023). Detailed chloride budgets in some DRB subwatersheds showed increasing storage of chloride as a result of long-term deicer applications as well (Rossi et al., 2022). Finally, a recent modeling study that leveraged the continuous SC sensor network in the DRB showed daily SC values primarily were driven by urban land use (Smith et al., 2024).

None of these studies, however, compared chloride concentrations to regulatory benchmarks in the DRB. This study fills that gap using data from 82 continuous non-tidal stream SC monitoring sites that span diverse ecological, geological, climate, and land use settings in the DRB. Establishing SC-chloride surrogate models at the continuous SC sites was a natural first step to characterize chloride exceedance events, but many of the continuous SC sites did not have discrete measures of chloride to establish those surrogate relationships. To address this, we used a cluster analysis to group sites based on watershed characteristics and pooled discrete chloride data for each cluster to develop cluster-based regression models. Site-specific and cluster-based regression models were used to predict hourly and daily mean chloride at the 82 sites for a three-year period (2020–2022). These chloride predictions were then compared to regulatory benchmarks. Finally, we explored the utility of SC as a predictor of chloride across a wide range of land use, climate, and geological settings.

## Methods

### Study setting

**Fig. 1:**
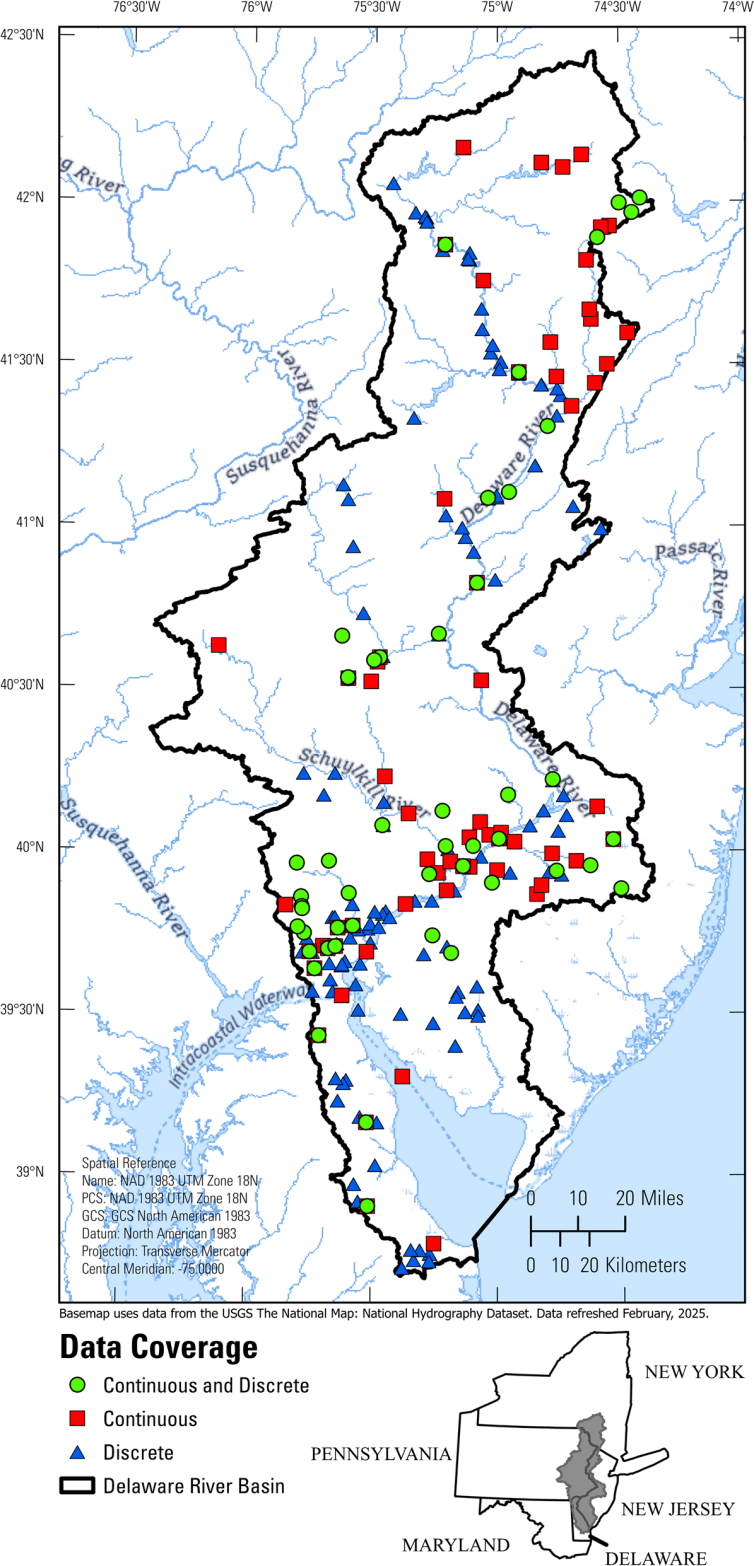
Map of the 209 monitoring locations within the Delaware River Basin (DRB) used for the study. Sites with continuous data collection (n = 95, of which 82 were non-tidal) were equipped with sensors that measured specific conductance (SC) at regular intervals (*e.g.*, hourly). Sites with discrete data coverage (n = 156) were measured at more infrequent intervals (weekly, monthly, or quarterly). Both continuous and discrete data were available at 42 sites. Fifty-three sites had continuous data available, and 114 sites had discrete data available. Base map hydrography scale = 1:100,000

The DRB encompasses approximately 40,000 km^2^ across New York (NY), Pennsylvania (PA), New Jersey (NJ), and Delaware (DE; Fig. 1) and spans diverse geological, ecological, climate, and land use/land cover (LULC) settings (Fig. SI-1). The northern part of the basin is underlain by sedimentary rocks, much of which is overlain by glacial deposits (Horton, 2017; Soller et al., 2009). In Northern NJ and PA, the Valley and Ridge Province (Fenneman & Johnson, 1946) consists of folded sandstone, shale, and carbonate rocks, some with karst features (Trapp & Horn, 1997). Further south and east, a section of the New England Province, consisting of crystalline and metamorphic bedrock, separates the Valley and Ridge from sandstones and shale in both PA and NJ. Metamorphic Piedmont rocks are present in southeast PA and northern DE. Unconsolidated sediments form the surficial units of the Coastal Plain Province. Large LULC gradients exist across the DRB with forests concentrated in the north, and suburban and urban LULC concentrated in the south (Dewitz and U.S. Geological Survey, 2021). Agriculture LULC is dominant in the NJ and DE Coastal Plain subregions, although it also is common in and around carbonate areas. Agriculture in the region is a mix of pasture and row crops, such as corn and soybeans. Climate and elevation vary across the DRB, with a larger percentage of annual precipitation falling as snow in the northern part of the basin (Wieczorek et al., 2018).

### Compiled datasets

Three datasets were compiled for this analysis: 1) SC data collected at continuous-monitoring stations in the DRB used to generate chloride predictions; 2) discrete measurements of SC and chloride used to develop SC-chloride regression models; and 3) watershed characteristics used to conduct the cluster analysis and other exploratory analyses. The first dataset was comprised of SC data from all continuous USGS sites in the basin (95 in total, including 82 non-tidal sites; Fig. 1) for water years 2020–2022 (October 1, 2019 to September 30, 2022), which was downloaded using the *dataRetrieval* package in R (De Cicco et al., 2023; version 4.2.1). SC was measured at these sites at high-frequency intervals (5 to 60-min intervals) using in situ sensors, thereby providing “continuous” measurements of SC. SC was measured using YSI™ or In-Situ™ SC sensors (see SI for more information). Established USGS procedures for sensor calibration, deployment, maintenance, and data review and approval and quality assurance were followed at all continuous SC sites (Wagner et al., 2006).

We compiled a second dataset of discrete measures of chloride and SC as a subset of a previous data compilation effort for the DRB (Shoda et al., 2019). These data originated from the National Water Quality Portal and had undergone several data cleaning and harmonization steps. The Shoda et al. (2019) dataset was queried to identify sites containing at least eight paired measurements of discrete chloride and SC from January 2010 to February 2022 (8 paired measurements was estimated as the minimum number to develop a linear regression between SC and chloride; see SI for more details). We identified 156 sites that fit the discrete paired measurement criteria in the DRB (Fig. 1).

All study sites were linked to flowlines in the National Hydrography Dataset (NHD) Plus Version 2.1 (NHDPlusV2.1; 1:100 K scale) network (McKay et al., 2012), which is a geospatial framework representing stream and river flowlines. Site location coordinates were used to join the sites to the NHD flowline network and assigned an initial reach ID, an identifier for NHD flowlines. Reach IDs then were visually inspected to verify sites were linked to the correct NHD flowline and were manually corrected, if necessary. Once all study sites had a reach ID, the presence of co-located continuous and discrete sites were assessed following protocols established by Murphy and others (2020; see SI for more details). Forty-two of the 95 continuous SC sites (44%) had discrete paired measures of chloride and SC collected at the same location or at a nearby co-located site. The other 53 continuous sites had few or no paired SC-chloride measurements. The remaining 114 of the 209 sites had discrete measurements only (Fig. 1).

Once all sites were linked to the NHD flowline network, we compiled a set of 50 watershed characteristics to describe LULC, geology, potential contaminant sources, physical settings, and climate for all study sites (Table SI-1). Watershed boundaries for sites were first delineated in ArcGIS™ (Environmental Systems Research Institute, Inc). Raster datasets for National Land Cover Dataset (NLCD) LULC (Dewitz and U.S. Geological Survey, 2021), major lithology (Horton, 2017), and surficial lithology (Soller et al., 2009) were used to compute percentage coverages for each watershed. We acquired the remaining characteristics from previously published datasets that were summarized for the upslope accumulated area for each NHDPlusV2.1 reach ID. Variables describing impervious surface cover (ISC), riparian LULC, lithologic geochemistry, mine density, and point source density characteristics were acquired from U.S. EPA’s Stream Catchment (StreamCAT) database (Hill et al., 2016). Variables describing percentage of mining, road networks, and climate characteristics were acquired from a U.S. Geological Survey database (Wieczorek et al., 2018). Details on variable names, definitions, and dataset sources can be found in Table SI-1. The compiled watershed characteristics, and the other input datasets, can be found in the associated data release (Hardesty et al., 2025).

### Cluster analysis

All study sites (continuous and discrete) were assessed for tidal influence by examining water quality data at each site and location relative to the marine salt front in the mainstem Delaware River or proximity to Delaware Bay (see SI for more details). A cluster analysis, based on watershed characteristics, was used to group the non-tidal sites. Fifteen sites (six continuous sites and nine discrete sites) were identified as tidally influenced and were separated into their own group because the watershed characteristics dataset did not reflect their primary source of salinity. We first conducted a principal component analysis (PCA) with the watershed characteristics to reduce dimensionality (Jolliffe, 2010). The PCA was conducted using the ‘prcomp’ function in the R *stats* package after data were centered and scaled (R Core Team, 2022). The PCA scores from the top five loading principal components were selected for cluster analysis and were assessed for their cluster tendency using the Hopkins statistic (Hopkins & Skellum, 1954). Next, hierarchical agglomerative cluster analysis (HACA) was performed. HACA is a bottom-up approach where each site begins as an individual group and linkages are determined based on similarity in their principal component values (Arora & Keshari, 2021; Fleming et al., 2013; Juahir et al., 2011). Euclidian distances were computed from the PCA scores using the ‘dist’ function from the *stats* package and the cluster analysis was conducted using Ward’s minimum variance method (Ward, 1963) with the ‘hclust’ function from the *stats* package. We evaluated different numbers of clusters based on how well they achieved two project goals: 1) generate enough clusters to represent the diverse geological, LULC, and climate settings across the DRB, and 2) ensure the 53 continuous sites that lacked discrete chloride data were grouped with discrete sites.

### Site-specific and cluster-based surrogate regression models

Site-specific regression models were developed for all 156 discrete sites using paired measures of discrete SC (explanatory variable) and chloride concentration (response variable). Regression models also were developed for each cluster of sites by pooling all discrete data from the sites within a cluster. Data outliers were assessed and removed following methods from Moore and others (2020). Outliers were considered for removal based on leverage, Cook’s distance, and standardized residual values (See SI for more details). Seventy-two outliers were removed from the 8,939 original discrete measurements (0.8% of the total dataset). We considered both linear and piecewise regression model forms for all sites and clusters. Piecewise regression was considered as an alternative to simple linear regression because chloride exhibits threshold responses at some sites (Moore et al., 2020), and piecewise regression can capture shifts in model slope. Linear regression models were generated using the ‘lm’ function from the *stats* base R package and piecewise regression models were developed using the ‘segmented’ function from the *segmented* package (Muggeo, 2008). Initially piecewise regression models were considered when their adjusted coefficient of determination (R^2^) was 0.02 or greater than the linear regression model, and there were at least three values on either side of the breakpoint. Next, all piecewise regression models were visually inspected, and segment slopes were assessed. Piecewise regression models were selected when the slope of the second segment was greater than the slope of first segment. In all other cases, the linear regression model was selected. Site-specific models were assigned to the 42 continuous sites with discrete data located at or near a continuous SC site. For the remaining 53 continuous sites, the relevant cluster-based regression model was assigned. Model regression forms and fit statistics for all 95 continuous sites are included in the associated data release (Hardesty et al., 2025).

### Data analysis using chloride predictions

Regression models were applied to the continuous SC datasets to generate chloride predictions at most continuous sites (88 of 95 sites). Seven of the 95 continuous sites were recently established and were included in the cluster analysis and regression model development. Their continuous SC data had not yet been reviewed and approved by time of publication, however and were therefore dropped from the remainder of the analysis. However, assigned regression models for these sites were included in the associated data release for future application. Chloride predictions at the remaining 88 continuous sites (82 non-tidal and 6 tidal) were reviewed. Predictions of negative chloride concentrations were replaced with a value equal to one-half the minimum discrete measured chloride concentration for the site or cluster. We next generated hourly and daily mean predicted chloride timeseries datasets for further analysis. Small gaps (*i.e.,* less than 24 h) in predicted chloride timeseries data were assessed. Days with less than 50% coverage were omitted from the daily timeseries dataset, and hours with any missing data (*i.e.*, a 15-min interval) were omitted from the hourly timeseries dataset. Daily and hourly mean predicted chloride concentrations for both non-tidal and tidal continuous sites can be found in the associated data release (Hardesty et al., 2025).

Predicted daily mean chloride concentrations at 82 non-tidal sites were summarized for seasonal and annual timescales. Seasons were defined as the following: Fall consisting of October and November; Winter consisting of December, January, February, and March; Spring consisting of April, May, and June; and Summer consisting of July, August, and September. Larger gaps (*i.e.,* greater than or equal to 24 h) were used to assess whether to report seasonal and annual values. For seasons, values were reported when 80% or more of the days had complete data (*e.g.*, 80% or more of spring). Exceedance probabilities for predicted daily mean chloride concentrations were computed for each season in water year 2021. Exceedance probabilities were computed by ranking the daily mean chloride predictions in descending order and dividing their rank by the count of daily mean chloride predictions within a season. For annual metrics, values were reported if 80% or more of days during winter had complete data (winter data gaps are listed in Table SI-2). Predicted daily mean chloride concentrations were averaged annually for water years 2020–2022.

### Characterizing exceedance events

The exceedance event analysis focused on the 82 non-tidal continuous sites with approved data for water years 2020–2022. We calculated exceedances of the EPA chloride chronic and acute criteria (EPA exceedance; 860 and 230 mg L^−1^, respectively) using predicted hourly and daily mean chloride concentrations. Chronic exceedance events were computed by generating a four-day moving average timeseries (right-aligned) from the daily mean predicted concentration timeseries. A chronic exceedance event occurred when the four-day rolling average was higher than 230 mg L^−1^. Acute exceedance events were assessed using hourly mean predicted chloride concentrations. An acute exceedance event occurred when hourly mean chloride concentrations were higher than 860 mg L^−1^. Start and end dates for individual exceedance events were compiled to generate a count of events at each site as well as duration above exceedance criteria.

Two sources of uncertainty were explored in the exceedance event analysis. First, is uncertainty in predicted chloride concentrations because of variability in the accuracy of SC-chloride regression models. To assess the effect of this uncertainty, we delineated exceedance events using the 5th, 50th, and 95th percentile predicted chloride concentrations (hereafter referred to as lower, median, and upper chloride predictions, respectively). Lower, median, and upper chloride predictions were generated using the R *stats* package ‘predict’ function (interval ="predict") on the original continuous SC data. These predictions were then averaged at hourly and daily timesteps and used in the exceedance analysis. Second, recent studies have suggested biological impairment may occur at chloride concentrations below EPA benchmarks, indicating the benchmarks may not be protective enough (Elphick et al., 2011; Miess & Dzialowski, 2024; Miltner, 2021). Therefore, we also used the Canadian Water Quality Guideline (CWQG; Canadian Council of Ministers of the Environment, 2011) as an additional benchmark to assess ecological risk. The chronic CWQG benchmark is 120 mg L^−1^ and serves as a more conservative threshold than the EPA chronic threshold (Weatherson et al., 2024). We identified sites that had at least one day above the CWQG and characterized uncertainty regarding the occurrence and duration of CWQG exceedances using the lower, median, and upper chloride predictions.

## Results

### Cluster analysis results

The top five principal components (PCs) cumulatively explained 57% of total variance in watershed characteristics across the dataset. LULC, including urban development and forest, had the highest loadings for the first PC (PC1), along with surficial lithology (Table SI-3). Unconsolidated major lithology (Coastal Plain) and wetland LULC loaded highly on PC2. Carbonate lithology, mining activities, and other LULCs dominated PC3, PC4, and PC5 respectively (Table SI-3). The PC scores for the first five PCs were evaluated for their clustering tendency using the Hopkins statistic (Hopkins & Skellum, 1954), which showed adequate clustering tendency (test value = 0.88; values above 0.5 indicate the dataset is suitable for clustering). We assessed cutoff values of six, eight, and ten clusters and visually inspected their watershed characteristics. Eight clusters plus the previously identified cluster of tidal sites (total clusters = 9) were optimal for the study goals in terms of generating interpretable clusters and pooling discrete sites with continuous sites (*i.e.,* no continuous sites were isolated in a cluster without discrete sites).

**Fig. 2:**
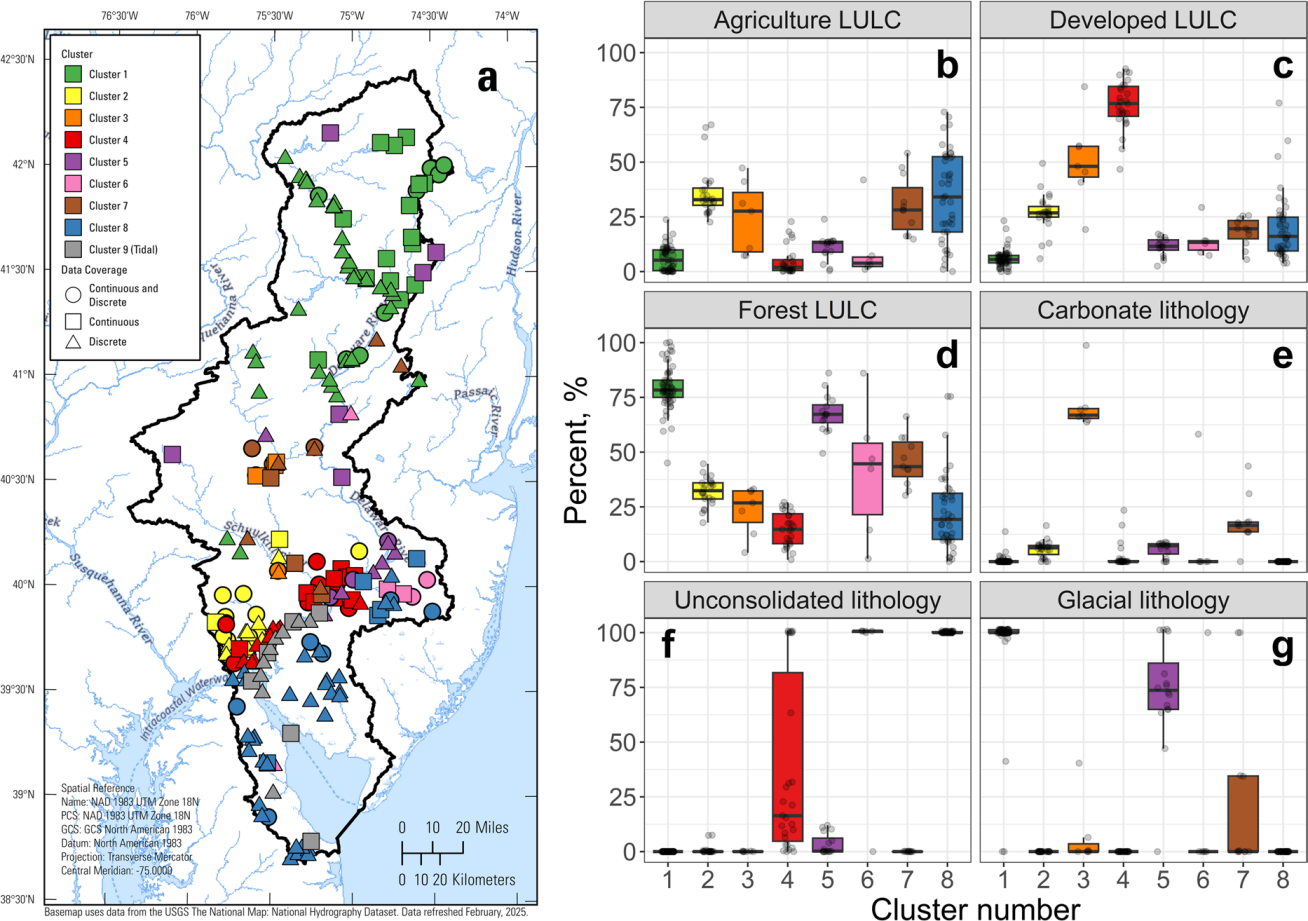
(**a**) Map showing cluster membership and site type for the 209 study sites in the Delaware River Basin. Colors correspond to the nine clusters, and symbology represents data coverage type (*i.e.*, continuous, discrete, or both; see text and Fig. 1 caption for details on site type). See Fig. SI-3 for maps showing individual clusters. (**b-g**) Box plots of watershed characteristics for sites within each of the eight non-tidal clusters, including (**b**) percentage of agricultural land use/land cover (LULC) (**c**) percentage of developed LULC; (**d**) percentage of forest LULC; (**e**) percentage of carbonate major lithology; (**f**) percentage of unconsolidated major lithology (*i.e.*, Coastal Plain); and (**g**) percentage of glacial surficial lithology. The middle horizontal line represents the cluster median of the variable on the y-axis, the box confines the 25th and 75th percentile, the tails represent one standard deviation. Points are jittered using the “geom_jitter” function in R (Wickham, 2016) to show the full spread of the data

The number of sites in the clusters ranged from 12 to 61 (Fig. SI-2), with spatial distinctions between many of the clusters (Figs. 2 and SI-3). Cluster 1 sites primarily were found in the northern third of the basin and had watersheds with high forest LULC and glacial surficial lithology. Sites in Cluster 2 primarily were located in the southern part of the basin and had watersheds comprised of mixed LULC underlain by metamorphic lithology. Cluster 3 included urban sites with watersheds underlain by carbonate lithology in or near Allentown, PA. Cluster 4 contained sites in watersheds with high urban LULC around Philadelphia, PA and Wilmington, DE. Cluster 5 primarily was composed of sites along the main stem of the Delaware River with no tidal influence. Cluster 6 had sites in watersheds with a high percentage of wetlands underlain by unconsolidated major lithology (*i.e.,* Coastal Plain). Sites in Cluster 7 had watersheds with mostly forested and agricultural LULC in watersheds underlain by bedrock. Cluster 8 represented sites in watersheds with mixed LULC in the Coastal Plain.

### Measured chloride concentrations and SC-chloride regression results

**Fig. 3:**
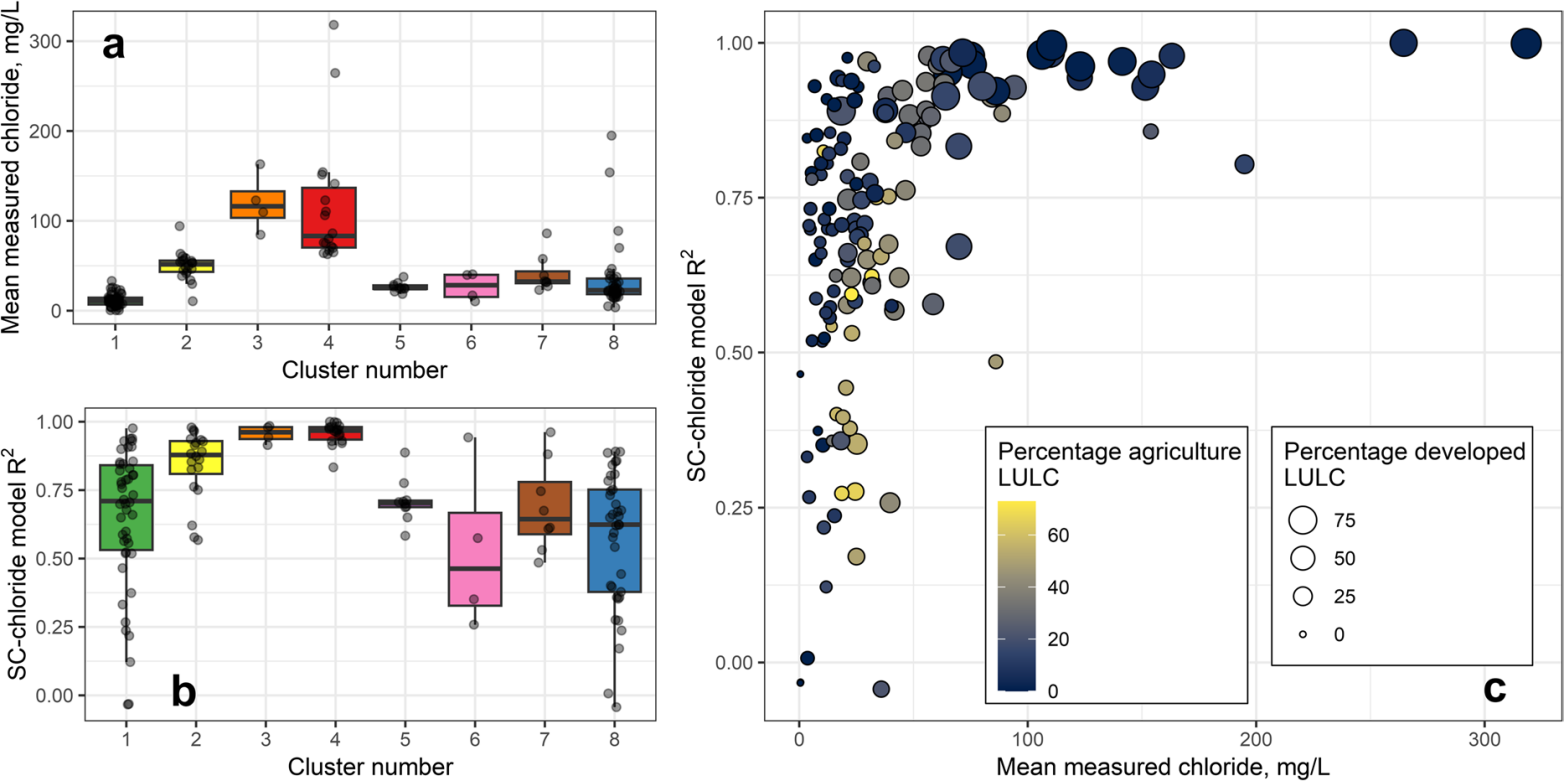
(**a**) Site means of measured chloride concentrations (mg L^−1^) for 147 discrete sites across the eight non-tidal clusters in the Delaware River Basin. (**b**) Model fits (adjusted coefficient of determination (R^2^) values) for site-specific specific conductance (SC)-chloride regression models for 147 sites. (**c**) The relationship between mean measured chloride concentrations and SC-chloride regression model fits for 147 sites. Color denotes the percentage of agricultural land use/land cover (LULC) and size indicates percentage of developed LULC in each watershed

Site means of measured chloride concentrations at the 147 non-tidal discrete sites varied from 0.5 mg L^−1^ to 318 mg L^−1^ with the lowest mean concentrations found in forested clusters (Cluster 1) and highest mean concentrations found in the urban clusters (Cluster 4; Fig. 3a). Site-specific SC-chloride regression model fits varied within, and among, the eight non-tidal clusters (Fig. 3b). Sites in clusters where developed LULC exceeded 25% (Clusters 2, 3, and 4) generally had the strongest model fits (predominantly R^2^ greater than 0.75), indicating SC was a robust predictor of chloride in these settings. Cluster 3, composed of urban sites in watersheds underlain by carbonate geology, had the least variability in SC-chloride regression model fits. Model fits were highly variable, and generally lower, in clusters dominated by forest, wetland, and agricultural LULC. Model fits were strong (R^2^ greater than 0.8) and mean concentrations exceeded 50 mg L^−1^ in highly urbanized sites (developed LULC greater than 75%; Fig. 3c). By contrast, model fits and measured chloride concentrations generally were lower at sites with high agricultural LULC in their watersheds. Site-specific estimates of slope from simple linear regression models (122 of the 147 sites) ranged from −0.05 to 0.33. Estimated slopes were generally greater than 0.2 in areas of higher developed LULC (e.g., Clusters 2, 3, 4; Hardesty et al., 2025).

Discrete measures of SC and chloride varied substantially among clusters (Figs. SI-4 and SI-5), with the highest values in urban clusters and lowest values in forested clusters (Clusters 1, 5, and 6; Fig. SI-4). The relationship (*i.e.,* slope) between SC and chloride varied among non-tidal clusters, and a wide range of chloride concentrations were associated with the same SC values across different clusters (Fig. SI-5). For example, a SC measurement of 300 µS cm^−1^ was associated with chloride measurements ranging from less than 20 mg L^−1^ to over 60 mg L^−1^. The resulting cluster-based SC-chloride regression models varied in terms of overall model fit and form (Table 1). Piecewise regression models yielded stronger fits for four clusters, including the three clusters with higher developed LULC (Clusters 2, 3, 4). The strongest model fits were for Clusters 3 and 4, respectively. The model for Cluster 4 also had the highest slope of non-tidal clusters (0.214 for the first segment and 0.331 for the second segment). Weaker model fits occurred in clusters with relatively low chloride concentrations in non-urban settings (*e.g.*, Clusters 5 and 6, Figs. 3a, b). For example, Cluster 6—which is dominated by forested wetlands—had an adjusted R^2^ of 0.55.

**Table 1:** Regression results for the nine cluster-based SC-chloride models that were applied to the 53 continuous SC sites lacking discrete chloride data in the Delaware River Basin

Cluster number	Number of continuous sites in cluster	Minimum measured Cl, mg L^−1^	Maximum measured Cl, mg L^−1^	Final model form	Segment 1		Segment 2		Breakpoint	Model adjusted R^2^
					Slope	Intercept	Slope	Intercept		
1	15	0.2	52	Linear	0.16	−0.7	NA	NA	NA	0.67
2	4	7.2	409	Piecewise	0.14	−0.6	0.33	−83.0	430	0.88
3	3	61.5	900	Piecewise	0.13	7.0	0.35	−160.9	749	0.99
4	9	5.0	1310	Piecewise	0.21	−12.3	0.33	−117.6	906	0.96
5	6	7.0	109	Piecewise	0.12	0.9	0.20	−19.1	243	0.79
6	2	1.4	137	Linear	0.08	9.7	NA	NA	NA	0.55
7	3	5.9	233	Linear	0.13	−10.2	NA	NA	NA	0.65
8	5	2.8	607	Linear	0.17	−8.9	NA	NA	NA	0.89
tidal	6	11.0	6150	Linear	0.32	−64.0	NA	NA	NA	0.99

The number of continuous sites in each cluster is the number of sites where cluster-based models were applied to predict chloride. Clusters with piecewise regression models have slope and intercept values for the two linear segments, as well as the breakpoint that denotes the SC value where the second segment begins. NA = not applicable.

### Spatial and temporal patterns in predicted chloride concentrations

**Fig. 4:**
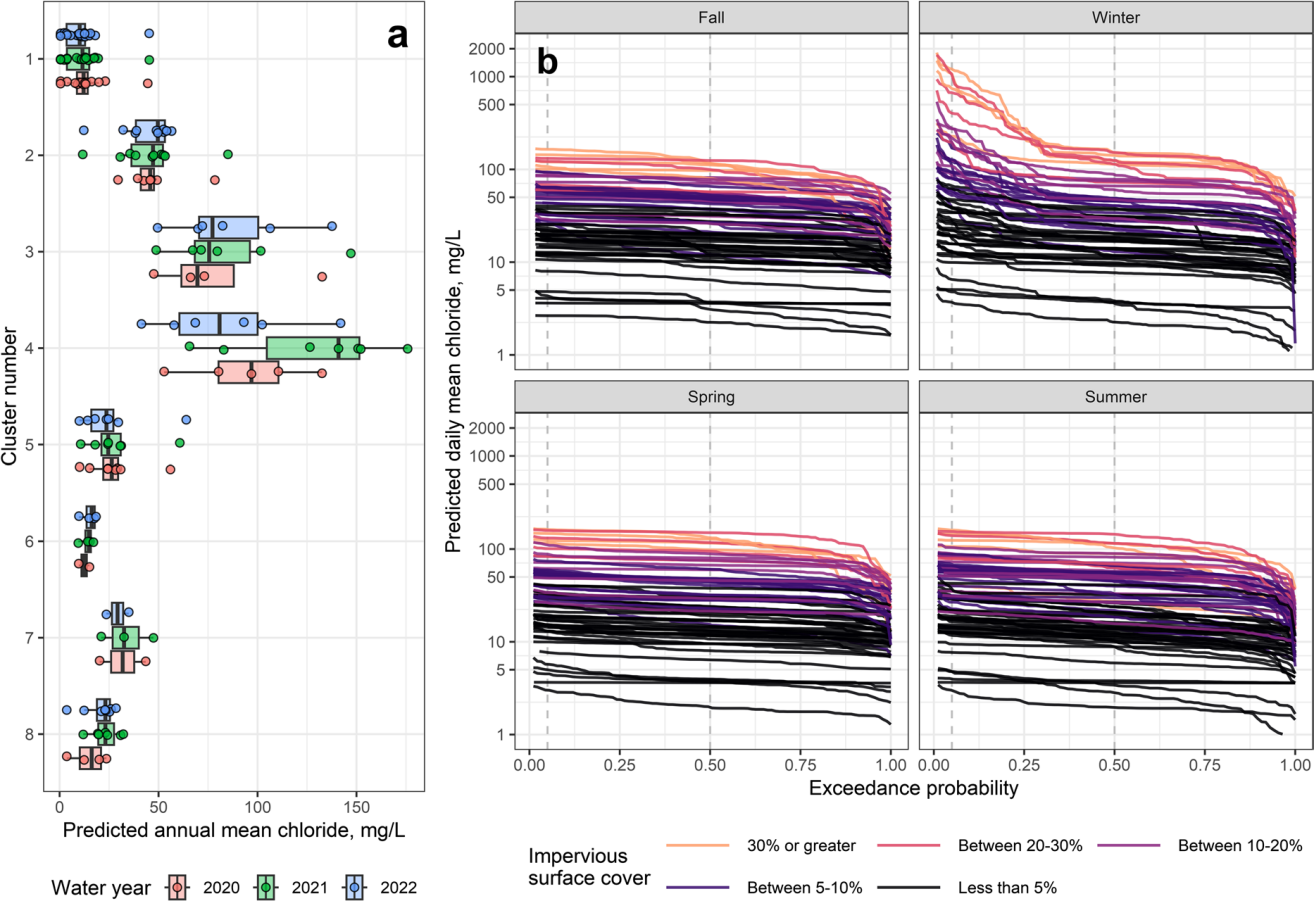
(**a**) Predicted annual mean chloride concentrations calculated from predicted daily mean chloride concentrations for non-tidal continuous sites (n = 48, 64, and 66 sites for water years 2020, 2021, and 2022, respectively) in the Delaware River Basin. Sites with large winter gaps (defined as more than 20% daily mean values missing for the winter season) were omitted from the plot. (**b**) Exceedance probabilities for predicted daily mean chloride concentrations for non-tidal continuous SC sites for the 2021 water year, broken into seasons (n = 76, 64, 74, and 78 sites for fall, winter, spring, and summer, respectively). Sites with large seasonal gaps (defined as more than 20% daily mean values missing for the season) were omitted from the plot. Higher predicted chloride concentrations have lower exceedance probabilities. Sites are colored based on the percentage area of the watershed with impervious surface cover. Vertical dashed lines denote the 0.05 and 0.50 exceedance probability chloride concentrations

Predicted chloride concentrations at the 82 non-tidal continuous SC sites varied spatially and temporally across the 2020–2022 study period (Fig. 4). As with the discrete chloride data, predicted annual mean chloride concentrations were highest in the urban clusters (greater than 75 mg L^−1^ in Clusters 3 and 4; Fig. 4a). By contrast, predicted annual mean chloride concentrations in Cluster 1 (forested) remained below 25 mg L^−1^ at all but one site. Clusters also exhibited different patterns in interannual variability. The highest predicted annual mean chloride concentrations occurred in 2021 at sites within Clusters 3 and 4. In Allentown and Philadelphia, where many Cluster 3 and Cluster 4 sites were located, the winter of 2020–2021 had more snowfall events and a greater winter severity index compared to the other two years in the monitoring period (Table SI-4). In non-urban clusters, predicted annual mean chloride concentrations did not vary as much across years (Clusters 1, 5, 6, and 7). Large winter data gaps precluded computation of predicted annual mean metrics for many sites (Table SI-2). Only 59% (48) of the 82 non-tidal continuous SC sites did not experience large winter gaps in 2020. The percentage of sites with sufficient winter season data coverage increased for the latter two years (75% and 80% of sites for 2021 and 2022, respectively).

Predicted daily mean chloride concentrations also varied across seasons within a single year (*e.g.,* 2021; Fig. 4b). Across all seasons, predicted daily mean chloride concentrations increased with greater ISC. Highest concentrations, and greatest variability in concentrations, occurred in the winter season at sites with greater ISC. This variability can be quantified using the difference between the 0.5 exceedance probability predicted daily mean chloride concentration, which represents the median predicted daily concentration for the season, and the 0.05 percentile predicted concentration that represents levels only rarely exceeded during the season (*e.g.*, concentrations that might occur immediately following a deicer application event). For example, Darby Creek near Darby, PA (22% ISC) had a winter season 0.05 exceedance probability concentration that was 533 mg L^−1^ above the seasonal median chloride (125 mg L^−1^, Fig. SI-6). Even sites with lower ISC (less than 10%) exhibited elevated and more variable winter chloride concentrations, albeit more muted (Fig. SI-6). The winter season 0.05 exceedance probability concentration at Schuylkill River at Landingville, PA (6% ISC) was almost double the predicted seasonal median concentration (91 vs 49 mg L^−1^).

The median of winter season (0.5 exceedance probability) predicted daily chloride concentrations increased with indicators of urbanization (*e.g.,* percentage developed, percentage ISC, number of road crossings; Fig. SI-7). Higher percentage mining LULC did not correspond with higher predicted median winter chloride concentrations. The highest predicted chloride concentrations were not associated with high (40% or more) agricultural LULC; these sites typically had relatively low predicted chloride concentrations (40 mg L^−1^ or less). Elevated predicted median winter chloride occurred across multiple lithological settings (*e.g.*, percentage carbonate, igneous, and metamorphic), so predicted concentrations were not associated with any single major lithology. Interestingly, higher annual percentage snow was not associated with higher chloride concentrations; rather, predicted median winter chloride concentrations declined with greater percentage snow. This decline is likely because of LULC differences observed in the DRB; most of the urban development is in the south and forest LULC dominates the snowier north (Fig. SI-1).

### Occurrence and duration of EPA and Canadian chloride exceedance events

**Fig. 5:**
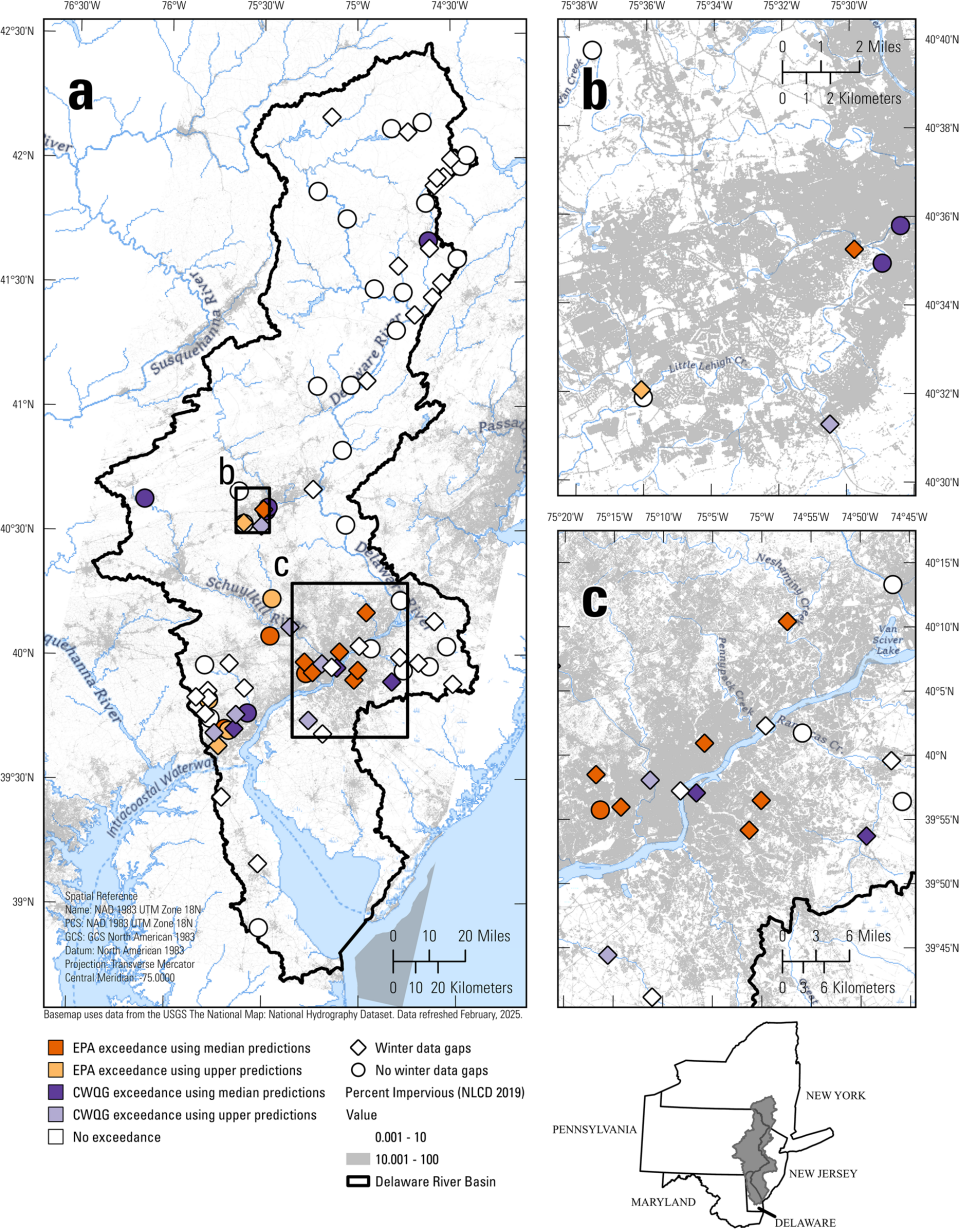
(**a**) Map of the 82 non-tidal sites in the Delaware River Basin showing the occurrence of chloride exceedances relative to the U.S. Environmental Protection Agency (EPA) chronic criterion (230 mg L^−1^) or Canadian Water Quality Guideline (CWQG) chronic criterion (120 mg L^−1^) using median and upper chloride predictions for years 2020–2022. Diamond shapes represent sites with substantial winter season gaps in the continuous data (*i.e.*, more than 20% of the 4-month period missing) for at least one of the three winter seasons. (**b**) Inset map showing the Allentown, PA region. (**c**) Inset map showing the Philadelphia, PA region

Predicted chloride concentrations exceeded EPA acute and chronic aquatic life criteria at numerous sites between 2020–2022 (Tables SI-5 and SI-6; Fig. 5). Ten sites had at least one chronic exceedance event when assessed using median chloride predictions (Table SI-5). Chronic exceedance events occurred at 5 additional sites (15 total) when the upper chloride concentration predictions were used. Each of the 10 sites with at least one chronic exceedance (using median chloride predictions) averaged three to four chronic exceedance events. Darby Creek near Darby, PA (DarbyCk, Cluster 4) experienced the most chronic exceedance events (8), and during these events predicted chloride concentrations exceeded the chronic threshold for 75 days in total. The median event duration at this site was 7 days (*i.e.*, 7 days when the 4-day rolling average was above 230 mg L^−1^ using the median chloride predictions).

Eight of 10 sites with chronic exceedance events also experienced acute exceedances (*i.e.*, predicted hourly mean chloride concentrations above 860 mg L^−1^ for one hour or longer; Table SI-6). An acute event was also flagged at a ninth site when upper chloride predictions were used. These sites experienced around 6–8 acute exceedance events each over the three-year period. South Branch Pennsauken Creek at Cherry Hill NJ (Spennsau) experienced the most acute exceedance events (11) with a total of 466 h spent above the acute exceedance threshold. All exceedance events occurred during the winter months (December through March). Given the number of sites that had at least one winter data gap (48 of the 82 sites; Fig. 5 and Table SI-2), the reported numbers may underestimate the extent and duration of chronic and acute exceedances in urban settings across the region.

More sites were flagged with chronic exceedances above the Canadian Water Quality Guideline (CWQG). Twenty-three sites had at least one day with predicted daily mean chloride concentrations at or above the 120 mg L^−1^ threshold when using median chloride predictions (Fig. 5; Table SI-7). Twenty-nine sites were flagged as exceeding the CWQG, at least once, when upper chloride predictions were used, and 21 sites were flagged as exceeding the CWQG, at least once, when lower chloride predictions were used. These sites were largely located in the central and southern part of the basin, especially around Allentown, PA (Fig. 5b), Philadelphia, PA (Fig. 5c), and Wilmington, DE (not shown).

**Fig. 6:**
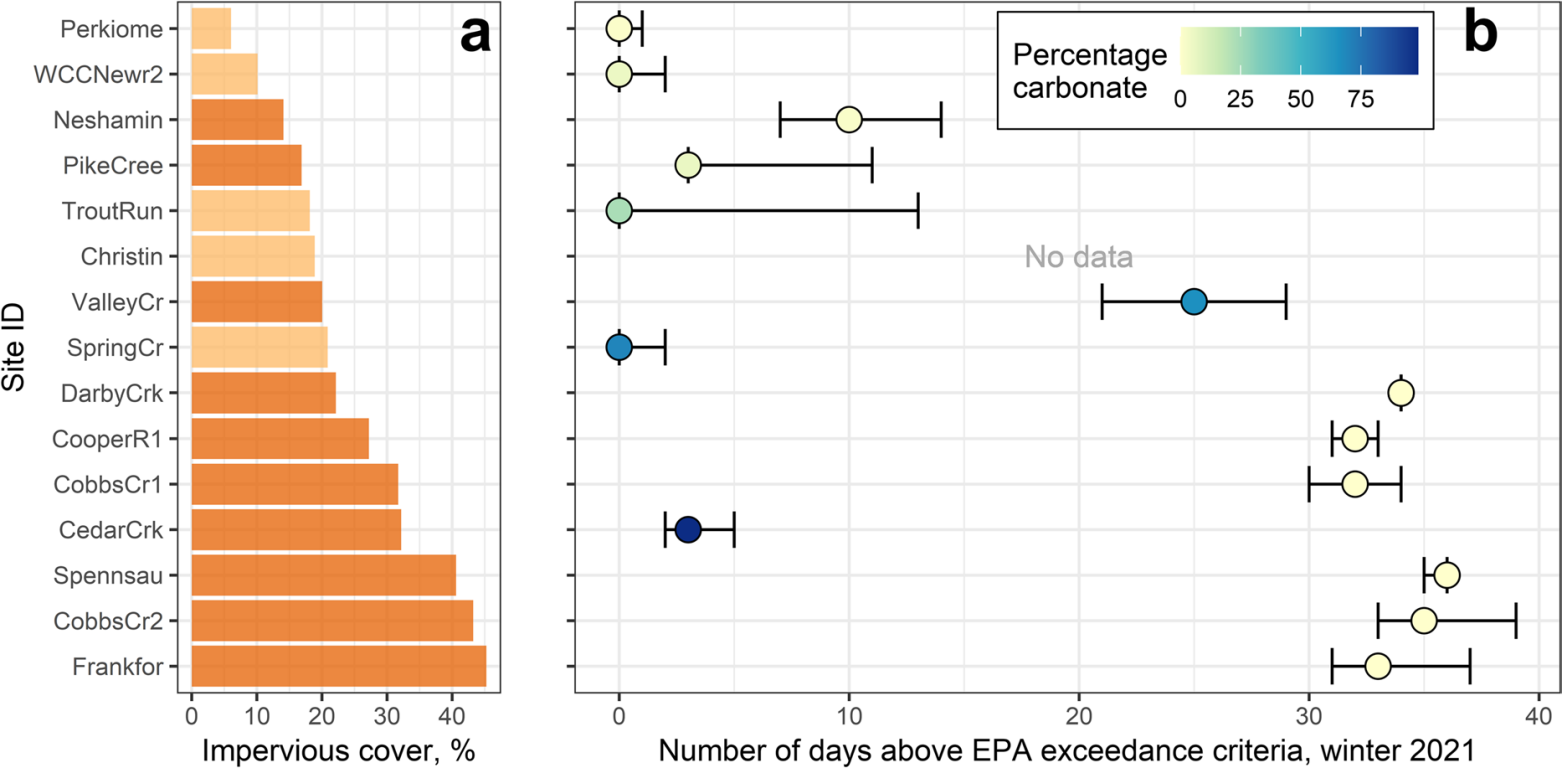
(**a**) Percentage impervious surface cover for the 15 sites in the Delaware River Basin that exceeded the U.S. Environmental Protection Agency (EPA) chronic criterion using median chloride predictions (dark orange) or upper chloride predictions (light orange). (**b**) Duration of chronic exceedances during winter 2021 (December 2020 through March 2021) for the 15 sites. Dots indicate the number of days the sites spent above the EPA chronic criterion using the median chloride predictions, and whiskers indicate the number of days the site spent above the EPA chronic criterion using the lower and upper chloride predictions. Dots are colored by the percentage of carbonate lithology

**Fig. 7:**
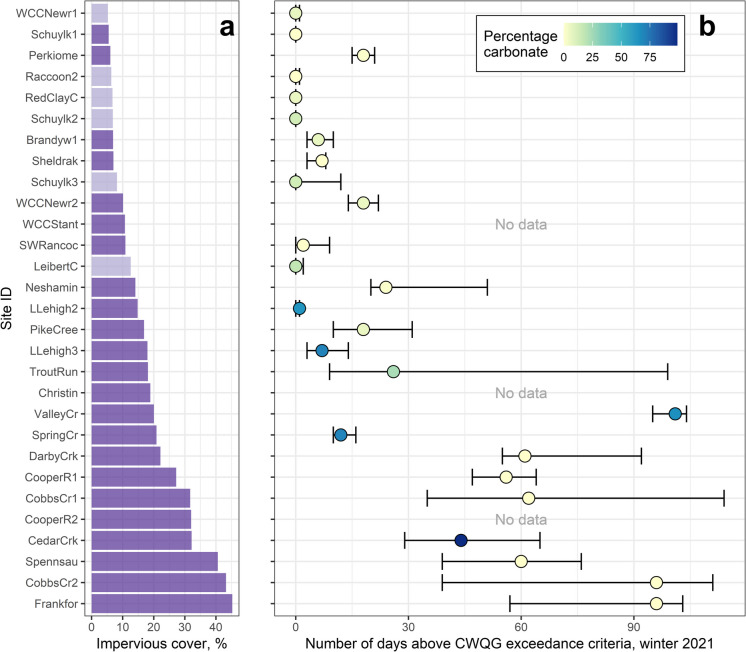
(**a**) Percentage impervious surface cover for 29 sites in the Delaware River Basin that exceeded Canadian Water Quality Guidelines (CWQG) chronic criterion using median chloride predictions (dark purple) or upper chloride predictions (light purple). (**b**) Duration of chronic exceedances during winter 2021 (December 2020 through March 2021) for the 29 sites. Dots indicate the number of days a site spent above the CWQG chronic criterion using median chloride predictions, and whiskers indicate the number of days the site spent above the CWQG chronic criterion using the lower and upper chloride predictions. Dots are colored by the percentage carbonate lithology

ISC for sites that exceeded the EPA chronic threshold ranged from 6–45% (Fig. 6a), and, for the 2021 winter season, the duration of chronic exceedance events generally increased with greater ISC (Fig. 6b). During that season, sites with less than 10% ISC rarely experienced EPA chronic exceedance events. Some sites with 10–20% ISC experienced exceedance events but had short exceedance durations (less than 10 days total). Most sites above 20% ISC experienced lengthy exceedance durations (20 days or more). Two exceptions were Spring Creek at Trexlertown, PA (SpringCr) and Cedar Creek at Mouth near Allentown, PA (CedarCrk), both of which are underlain by carbonate lithology (Fig. 6b). Exceedances of the CWQG occurred at sites with ISC as low as 5% and exceedance duration for winter 2021 also increased with greater ISC (Fig. 7). As expected, sites spent more time above the CWQG threshold than the EPA threshold. Uncertainty regarding the duration of exceedances was overall greater when the CWQG was applied.

**Fig. 8:**
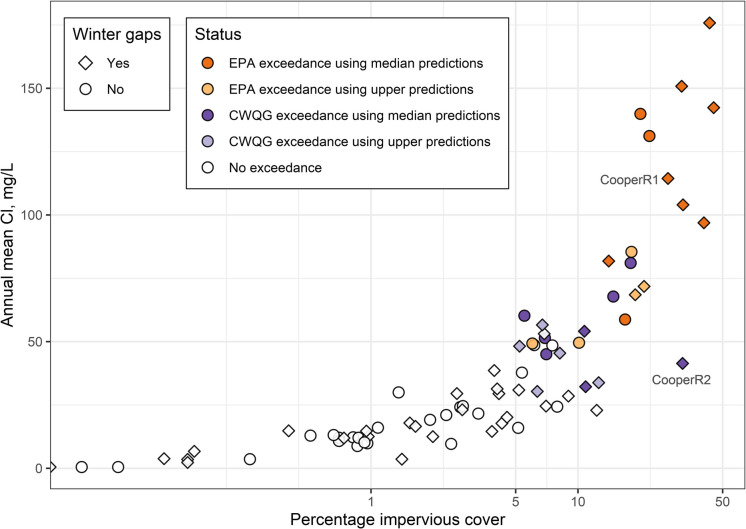
Percentage impervious surface cover versus predicted annual mean chloride (Cl) concentrations calculated from predicted daily mean chloride concentrations for 2020–2022 for the 82 non-tidal continuous SC monitoring sites in the Delaware River Basin. Sites where chloride exceeded the U.S. Environmental Protection Agency (EPA) water quality criteria or Canadian Water Quality Guidelines (CWQG) chronic criteria are denoted by their color. Diamond shapes represent sites with a substantial data gap during the winter season (greater than 20% of the season with missing data) for at least one of the three winter seasons. An alternative version of this figure using median annual chloride concentrations can be found in Fig. SI-8

Across the three-year monitoring period, higher predicted annual mean chloride concentrations were associated with greater ISC (Fig. 8). At low levels of ISC (less than 5%), mean chloride concentrations generally were well under 25 mg L^−1^. Predicted annual mean chloride ranged from 25–50 mg L^−1^ at sites with 5–10% ISC, and about half of them approached or exceeded the CWQG chronic criterion. Almost all sites with 10% or more ISC had annual mean chloride concentrations exceeding 50 mg L^−1^. One exception was Cooper River at East State Street at Camden, NJ (CooperR2), which has very high ISC (32% ISC) but relatively low predicted annual mean chloride concentrations (41 mg L^−1^). This location is close to the confluence of the Cooper River and the mainstem of the Delaware River in NJ. Streamflow data for CooperR2 indicated this site was influenced by backwater from the Delaware River that likely resulted in mixing of water with lower chloride concentrations from the Delaware River. Another continuous SC site located further upstream along the Cooper River (Cooper River at Haddonfield NJ; CooperR1) had lower ISC (27% ISC), but a much higher predicted annual mean chloride concentration (114 mg L^−1^), providing further evidence that chloride concentrations at the CooperR2 site likely were influenced by backwater. Three other sites with 10% or more ISC also had unusually low predicted annual mean chloride concentrations: 1) Southwest Branch Rancocas Creek at Medford NJ (SWRancoc; 11% ISC, 32 mg L^−1^); 2) St. Jones River at Dover, DE (StJonesR; 12% ISC, 23 mg L^−1^); 3) and Leibert Creek at Emmaus, PA (LeibertC; 13% ISC, 33.8 mg L^−1^). All three of these sites have monitoring gaps during the winter season, which likely resulted in lower predicted annual mean chloride. Two of these three sites also are located in the extreme southern part of the basin and receive somewhat lower amounts of snow and other frozen precipitation, and subsequent deicer applications.

## Discussion

### Patterns of ecological exceedance events and elevated chloride

Assessing risk of ecological impairment caused by freshwater salinization can be done most effectively with continuous water quality monitoring because it can capture short-duration events that are especially harmful to biota (Moore et al., 2020). Our study used continuous data to assess whether regulatory aquatic life benchmarks for chloride were exceeded across a temperate region with diverse LULC and geologic settings. About 35% (29) of 82 non-tidal sites in the continuous specific conductance (SC) network experienced exceedances above regulatory benchmarks, while 31% (25) of sites experienced no exceedances. Although we did not observe exceedances at the remaining 34% (28) of sites, prolonged data gaps during winter may have missed periods of elevated chloride (Figs. 5 and 8).

Exceedances of EPA water quality criteria for chloride rarely occurred in watersheds with less than 10% impervious surface cover (ISC), sometimes occurred in watersheds with 10–20% ISC, and regularly occurred in watersheds with 20% or more ISC (Fig. 6). EPA chronic exceedance events lasted up to about 30–35 days during winter 2021. These results are consistent with other studies that used a similar approach to quantify ecological exceedances with continuous data. For example, Moore et al. (2020) found exceedance events occurred in watersheds with at least 9–10% ISC in the mid-Atlantic and Northeast US regions. Median annual duration of exceedance events was 24 days in the study’s mid-Atlantic sites with similar levels of ISC. In the Northeast sites, however, annual durations of exceedance events were much higher, often lasting over 180 days. In another study based in Ontario, Canada, EPA chronic chloride exceedance events lasted 18 days or longer in watersheds with at least 15% ISC (Weatherson et al., 2024). Chloride concentrations exceeded the CWQG chronic criterion in DRB watersheds with as little as 5% ISC and exceedances lasted for most of the winter season in some cases (Fig. 7). Chloride concentrations remaining above regulatory benchmarks for extended periods of time may impact critical life stages of freshwater aquatic organisms (Lawson & Jackson, 2024).

Elevated chloride predictions at seasonal (Fig. SI-6) and annual scales (Fig. 8) were evident in watersheds with less than 5% ISC, indicating impacts to water quality can occur at very low levels of ISC even if chloride concentrations did not exceed regulatory benchmarks. Evidence of elevated and increasing salinity at very low levels of ISC (less than 5% ISC) have been documented in the mid-Atlantic region, especially in areas with seasonal snowfall (Baker et al., 2019; Fanelli et al., 2024). Elevated chloride across non-winter seasons was prevalent in urban settings as well. In fall, spring, and summer, predicted chloride concentrations were persistently higher in watersheds with greater ISC (Fig. 4b). Elevated salinity in non-winter months has been documented in other studies (Corsi et al., 2015; Lawson & Jackson, 2024; Mazumder et al., 2021), further highlighting this pattern in urban temperate regions. A recent trends study in the DRB proposed two processes likely driving freshwater salinization: 1) surface transport during winter seasons (*i.e.*, winter runoff pulses) and 2) subsurface accumulation and transport during non-winter seasons (*i.e.*, legacy contributions; Rumsey et al., 2023). Our study revealed winter runoff pulses throughout the continuous SC network at all levels of ISC, although the magnitude of these pulses increased with greater ISC (Fig. SI-6). Additionally, legacy contributions during non-winter seasons were especially evident in urbanized watersheds within our study (Fig. 4b). Indeed, a mass balance conducted in some of the urban study watersheds revealed chloride often was retained on an annual timescale, highlighting long-term storage of chloride in the subsurface across the region (Rossi et al., 2022).

### Winter snowfall patterns and deicer salt applications

Although ISC was the primary driver of elevated chloride, other factors likely impacted chloride patterns in DRB streams. Variability in winter weather patterns and deicer application timing, rates, and types likely influenced exceedance patterns in the DRB. Interestingly, the percentage of annual precipitation comprised of snow was inversely related to median winter-season predicted chloride concentrations (Fig. SI-7) that likely was driven by differences in urban development and climate. Most population centers and urban land cover in the DRB are in the central and southern parts of the basin (Fig. SI-1), where snowfall is relatively low. Elevated chloride is rare in the upper part of the basin (Fig. 5) despite more snow because of relatively low levels of development. Subsequent melting of larger snowfall amounts may also increase dilution of deicer applications as well.

The winter season for 2021 was more severe than the other two years in the monitoring period (Table SI-4). Snowfall for the season ranged from 18–56 mm (mm) across the counties where exceedances were documented (Table SI-8; Pennsylvania Department of Transportation, 2021) and likely influenced the amount of deicer applied. For example, Lehigh County, PA, which encompasses the city of Allentown (Fig. 5b), received approximately 56 mm of snow and used approximately 8.9 million kg of rock salt and 1.1 million liters of brine for deicing in winter 2021 (Table SI-8). Urban sites in this area (SpringCr, LLehigh3, CedarCrk, and LLehigh2) experienced five or fewer days above the EPA chronic exceedance criterion (Table SI-5; Fig. 6). By contrast, Delaware County, PA, which encompasses part of Philadelphia (Fig. 5c), received only 18 mm of snow and relied heavily on rock salt (applied about 22.4 million kg of rock salt and 225,000 L of brine) during winter 2021 (Table SI-8). Urban sites in this county (DarbyCrk, CobbsCr2) had durations of EPA chronic exceedances lasting over 30 days. Application of brine as an anti-icer (*i.e.*, before storm treatment) or deicer can result in 30–50% less salt use compared to rock salt (Fay et al., 2013; Michael Fitch et al., 2013). Brine application resulted in an average of 45% lower chloride loads in the days after salting (Haake & Knouft, 2019).

Differing deicing practices (*e.g.*, the use of brine vs. rock salt) likely contributed to some variability in exceedance event occurrence and duration among watersheds with similar percentages of ISC. Most brine applications occur on roads, however, and non-road ISC (*i.e.*, parking lots), which typically are only treated with rock salt, are substantial contributors to chloride inputs in the region (Rossi et al., 2022). As a comparison, private (non-government) deicer application to parking lots was estimated as 38% of total salt application in a dense urban watershed in Toronto, Canada (Perera et al., 2010). Many additional factors, including storm-specific road temperatures, weather forecasts, and anticipated vehicle density determine treatment options (Du et al., 2022) and influence the mass of brine or rock salt being applied.

Additional sources of chloride in urban settings, such as wastewater treatment plants and industrial discharges, may also contribute to elevated chloride in streams and rivers (Kaushal et al., 2023). Chloride inputs from these sources are typically greatest in late summer and fall when regional baseflow is lowest and those sources contribute a larger percentage of streamflow. Given the prevalence of exceedance events during the winter season, it is likely that seasonal deicer applications are primarily responsible for causing chloride exceedances in the region.

### Groundwater and geological influences on stream chloride dynamics

Chloride was elevated during non-winter seasons in urban streams within the study area (Fig. 4b) indicating accumulation and storage of chloride in the subsurface. Excess rock salt left on or near roadways may contribute chloride long after the winter event is over, and chloride from deicer material can be retained in surface soils for several months after application (Robinson et al., 2017; Rossi et al., 2017). Shallow groundwater may become contaminated with chloride from recharge beneath stormwater management practices, like detention ponds (Snodgrass et al., 2017), or beneath floodplains that may be released into the channel later in the season as baseflow (Ledford & Lautz, 2015). Winter recharge often may load chloride into deeper groundwater that also provides non-winter baseflow (Van Meter & Ceisel, 2024). In a mid-Atlantic urban watershed, some stream reaches had consistently high baseflow chloride concentrations across multiple years of synoptic sampling, and high concentrations correlated with groundwater flow paths originating in areas of higher ISC (Welty et al., 2023). Groundwater chloride concentrations approaching or exceeding the EPA chronic criterion have been documented across multiple geological settings (including within the DRB) as a result of subsurface accumulation (Lindsey et al., 2023; Van Meter & Ceisel, 2024; Weatherson et al., 2024). This slow release of stored chloride in the subsurface may delay responses to management practices implemented to reduce chloride loading to streams (Gutchess et al., 2018).

Geological setting influences groundwater flow paths, subsurface mixing, and residence times (Maxwell et al., 2016), which could affect chloride dynamics in streams. While elevated chloride concentrations and exceedances were observed across multiple geological settings (Fig. SI-7), shorter exceedance durations were observed at some sites underlain by carbonate lithology (Figs. 6b and 7b). Carbonate lithology often is associated with well-connected surface and groundwater systems and greater baseflow contributions to total streamflow compared to other geological settings. These baseflow contributions can result in both larger groundwater contributions to streamflow and smaller peak flows during runoff events (Le Mesnil et al., 2021). Mixing of relatively low chloride groundwater with winter season runoff during and immediately after runoff events may result in smaller chloride peaks and shorter durations of exceedance events in streams. A catchment modeling study in a carbonate watershed showed that while the volume of young water (*i.e.*, event water) increased during runoff events, discharge of old water also was quite high (Zhang et al., 2020), which could dilute the young water geochemical signal. This mechanism may explain the shorter exceedance durations observed in the urban carbonate sites (Figs. 6 and 7). However, elevated and highly variable chloride concentrations have also been documented in urban springs underlain by carbonate lithology (Robinson & Hasenmueller, 2017). We observed similar patterns at Valley Creek at Wilson Road near Valley Forge, PA (ValleyCr), suggesting that more research is needed in carbonate systems to better understand retention and transport of chloride in these settings.

### Site and cluster SC-chloride regression models

SC has been previously documented as a reliable predictor of chloride (Haq et al., 2018; Moore et al., 2020; Weatherson et al., 2024), and other major ions and metals (Galella et al., 2021). Site-specific SC-chloride regression model strength (*i.e.*, R^2^) and model slopes varied across the DRB, emphasizing variable contributions of chloride to measures of SC and uncertainty in predicting chloride using SC as the sole proxy. Model performance was most robust in areas of highest concern, namely, sites with moderate and high ISC, indicating that chloride is the primary driver of stream SC in areas with high ISC. Models at sites with mixed LULC (*i.e.*, both development and agriculture), often located in the Coastal Plain (Cluster 6), had lower slopes and lower R^2^, indicating additional ions, like nitrate, are likely contributing to SC in these areas. Regression models for forested sites also often had low slopes, indicating chloride was not a dominant ion in the system.

We documented variability in detection of exceedance events above regulatory benchmarks from uncertainty in the SC-chloride regression models. The number of sites with at least one EPA chronic exceedance event increased from 10 to 15 when the upper (95th percentile) chloride predictions were used (Table SI-5). However, results did not change when we used the lower (5th percentile) chloride predictions, indicating the analysis could be underestimating the occurrence of chronic exceedance events. Regression model uncertainty also resulted in variability in the duration above regulatory benchmarks (*e.g.*, Table SI-7). For example, chloride concentrations at Frankford Creek at Castor Ave, Philadelphia, PA (Frankfor) were predicted to exceed the CWQG benchmark anywhere from 277 to 602 days over the three-year period. The prediction intervals during baseflow conditions at this site sometimes overlapped with the CWQG benchmark, resulting in a large range in exceedance duration (Fig. SI-9). This pattern was most evident at Trout Run at Avondale, PA (TroutRun), where days at or above the CWQG benchmark ranged from 9–900. The upper chloride predictions at this site exceeded the CWQG for the majority of 2021 (Fig. SI-9). The SC-chloride model fit at Trout Run also was lower than at Frankford Creek (adjusted R^2^ of 0.83 vs. 0.99, respectively) resulting in wider prediction intervals at Trout Run. We only explored SC as a proxy variable for predicted chloride in this study. Often, regression models to predict chloride may be improved by adding additional predictor variables, such as discharge or season (Rossi & Gyves, 2023). Increased sampling across the full range of SC may also improve model accuracy and reduce uncertainty in chloride predictions.

Our study highlighted the value of developing cluster-based regression models based on watershed characteristics to fill knowledge gaps in the absence of discrete measures of chloride at a site. Cluster analysis previously has been used to classify sites based on water quality results (Ghaemi & Noshadi, 2022; Güler et al., 2002; Juahir et al., 2011). The goal of our study was to group sites based on watershed characteristics so chloride could be predicted at sites that lacked discrete sampling. This approach also allowed us to test the hypothesis that patterns in chloride and SC-chloride models would be consistent across watersheds with similar characteristics. In general, we found similarities in chloride concentrations and model forms in clusters/settings where chloride is a concern, illustrating that this approach was effective for extrapolating to unmonitored sites.

Importantly, this study showed how chloride-SC relationships varied across different clusters and the environmental settings they represented. For example, the same SC value measured at sites across the eight non-tidal clusters were associated with a wide range of chloride concentration measurements (Fig. SI-5). This finding highlights the risk of over-simplifying SC-chloride relationships when a “one-size-fits-all” regression equation is applied across a region with differing geology and LULC characteristics. The cluster-based regression approach outlined in this study is an effective way to predict chloride across diverse settings where both natural and anthropogenic factors influence SC patterns. Regional-scale variability in SC was documented in another study in the nearby Chesapeake Bay watershed, for which a flexible benchmark was used to quantify departures from background SC (Fanelli et al., 2024).

Our study also documented within-cluster variability of chloride concentrations (Fig. SI-4), as well as model performance (Fig. 3b). This variability could originate from 1) cluster criteria not grouping sites sufficiently to account for all the differences among watersheds, or 2) the watershed characteristics selected for the study not sufficiently explaining all potential drivers of chloride and SC-chloride relations. We aimed to balance the number of clusters with the need to ensure they were also interpretable. Moreover, the use of cluster analysis to group sites based on watershed characteristics has advantages over strict spatial clustering as origins of elevated chloride may substantially vary across small spatial scales (*e.g.*, ISC; Fig. 5).

### Benchmarks for defining ecological impairment

We used the EPA chronic and acute criteria for aquatic organisms to assess potential effects on biota (230 mg L^−1^ and 860 mg L^−1^, respectively). For these criteria to be met, these concentrations should not be exceeded more than once within a three-year period (U.S. EPA, 1988). We also applied the chronic CWQG (120 mg L^−1^), which is about 50% lower than the EPA chronic criterion (Canadian Council of Ministers of the Environment, 2011). Some studies, however, have shown that neither the EPA nor Canadian benchmarks may be sufficiently protective for aquatic organisms in some settings, and that lower benchmarks are needed to protect the ecological integrity of streams. A state-wide analysis of Ohio biological stream survey datasets revealed a hazard effect concentration of 52 mg L^−1^ for chloride, which is the concentration at which 5% of benthic macroinvertebrate taxa become extirpated based on species sensitivity distributions (Miltner, 2021).

Another analysis quantified taxa-specific changepoints (*i.e.*, thresholds) across gradients of chloride concentrations for different northern ecoregions in the USA (Miess & Dzialowski, 2024). That study identified “high impact” chloride thresholds (*i.e.*, 75% taxa effected) at 30 mg L^−1^ and 75 mg L^−1^ for the Southern Appalachian and Northern Appalachian ecoregions, respectively, which are the two ecoregions that represent the DRB region. The higher threshold in the Northern Appalachian ecoregion suggests taxa that are most salt intolerant may have already been extirpated in many streams. This may indicate that more sites within the DRB continuous SC network are at risk from chloride contamination than those flagged in this study.

Some sites located near Delaware Bay could also be impacted by sea salt aerosols that could increase background levels of chloride and only support taxa that are naturally more tolerant to higher levels of chloride. In these settings, ecological impairment may not be as severe as in naturally low-saline streams. Additional work quantifying concentrations of other ions, such as sodium or calcium, and ion mixtures relative to background or reference conditions is needed to fully understand the impacts of freshwater salinization on stream ecosystems (Clements & Kotalik, 2016; Cunillera-Montcusí et al., 2022).

### Continuous SC monitoring network in the DRB

Continuous water quality monitoring with in situ sensors has great value for both surface waters (Rode et al., 2016) and groundwater (Saraceno et al., 2018). Continuous water quality datasets often are used as proxies for water quality constituents of interest, like nutrients (Kermorvant et al., 2023; Villa et al., 2019), and chloride (Moore et al., 2020; Weatherson et al., 2024). The DRB continuous SC monitoring network provided a timely opportunity to explore the use of SC as a proxy for chloride and characterize high-frequency dynamics of predicted chloride concentrations across a wide range of settings. The network was originally established to address multiple stakeholder data needs within the DRB, including temperature, flow, and salinity (Eberts et al., 2019). SC monitoring was sometimes added as an ancillary parameter to flow or temperature monitoring sites, and as such, the SC network we leveraged for this study may not adequately represent areas within the DRB where chloride and freshwater salinization are a concern. The current network is comprised of sites mostly representing forested settings that are at low risk of chloride exceedances and contained fewer sites with chloride and SC conditions that are actively exceeding regulatory benchmarks (Fig. 8). For example, the median percentage ISC across the continuous SC network is 6%, and over 25% of the SC continuous sites have less than 2% ISC. Adjusting the monitoring network to include more areas that are at greater risk from freshwater salinization may be useful for future basin-scale assessments.

Gaps in continuous monitoring across the continuous SC monitoring network impacted our assessment. Only 36 sites had complete winter coverage for the study period and 18 sites had winter gaps during at least two of the three years (Table SI-2). Most continuous sites experienced short gaps (less than 24 h) in SC monitoring from sensor malfunction or maintenance. Some sites also experienced longer gaps in the winter season from the sensor being temporarily removed to avoid damage from ice development. Unfortunately, sensor removal in these cases also occurred when chloride likely was elevated from deicer applications, leaving monitoring gaps during critical times. Despite these unavoidable data gaps, the DRB continuous SC data provide valuable insights into the occurrence of chloride exceedances across diverse settings. Additional monitoring in urban areas, some of which is already underway, will help better characterize the extent and severity of freshwater salinization in the region.

## Conclusions

Documenting spatial and temporal extent of chloride exceedance above regulatory benchmarks in the Delaware River Basin (DRB) is a critical first step to mitigate the impacts from freshwater salinization. In our study chloride exceedances above regulatory benchmarks occurred in watersheds with 5% or greater impervious surface cover (ISC), but seasonally elevated chloride in watersheds with less than 5% ISC was also documented. In addition to ISC, other factors likely contributed to exceedance event characteristics and variability in chloride concentrations, including deicer material types, application rates and practices, and winter weather patterns. Geological settings and groundwater contributions might also have influenced stream chloride patterns, but more work is needed to understand these effects on chloride-impervious surface relationships (*i.e.*, Fig. 8). This study demonstrated the value of continuous water quality monitoring to characterize temporally dynamic conditions, and our results highlight the need to explore storage and release dynamics of subsurface contaminants like chloride. Our study confirmed SC served as a robust predictor for chloride in settings where chloride was the dominant ion from deicer applications. Cluster analysis was an effective way to transfer information to sites lacking discrete water quality data and may be used for development of other surrogate models (*e.g.*, for sediment or nutrients). Moreover, this approach could be used in other regions with sparse data collection to fill water-quality-monitoring gaps for surrogate modeling (Villa et al., 2019). Finally, results can be used to identify reaches and settings where mitigation activities can be targeted to slow or reverse increasing trends in salinity observed throughout the region. Regional stakeholders in the DRB are implementing such practices, such as precision deicer applications, to reduce chloride loading to local waterways. Studies like this are critical for documenting progress from regional mitigation efforts, in addition to characterizing drivers of chloride dynamics in temperate streams and rivers.

## Supplementary Information

Below is the link to the electronic supplementary material.

Supplementary file1 (DOCX 7.27 MB)

Supplementary file2 (XLSX 24.3 KB)

## Data Availability

Data availability: All data used for, or generated in, the analyses, including discrete measures chloride and SC observations, watershed characteristics, final SC-chloride model regression equations, and daily and hourly chloride predictions, are available in the associated data release: Hardesty, D.M., Fanelli, R.M., Morency, M., 2025. Discrete chloride and specific conductance (SC) measurements, chloride-SC regression equations, and daily and hourly mean SC values and chloride predictions for 88 USGS water quality monitoring stations in the Delaware River Basin, 2019–2022: U.S. Geological Survey data release. 10.5066/P146TC5X.
