# Supplementary material for: Regional high-frequency monitoring revealed chloride concentrations in exceedance of ecological benchmarks in urban streams across the Delaware River Basin, USA
Source: Environ Monit Assess. 2025 Aug 29;197(9):1056. doi: 10.1007/s10661-025-14485-6 (PMC12394352; doi:10.1007/s10661-025-14485-6)
Supplement: Supplementary file 1 — Supplementary file1 (DOCX 7.27 MB) [file 10661_2025_14485_MOESM1_ESM.docx]

**Supplemental information** for “Regional high-frequency monitoring revealed chloride concentrations in exceedance of ecological benchmarks in urban streams across the Delaware River Basin, USA”

**Authors**: Rosemary M. Fanelli^1^*, Michelle Morency^1^, Brandon J. Fleming^2^, Joel Moore^3^, Deanna Hardesty^1^, and Megan Shoda^4^

^1^U.S. Geological Survey South Atlantic Water Science Center, Raleigh, NC, USA

^2^U. S. Geological Survey Pennsylvania Water Science Center, New Cumberland, PA, USA

^3^Towson University, Towson, MD, USA

^4^U.S. Geological Survey Water Mission Area, Reston, VA, USA

*Corresponding author (rfanelli@usgs.gov)

**Methods**

**Monitoring continuous specific conductance (SC):** During data collection for this project, the U.S. Geological Survey (USGS) employed several types of SC sensors across the continuous SC monitoring network:

1. In-Situ Aqua TROLL™ sondes (500 and 600 models) equipped with the Aqua TROLL™ Temperature-Conductivity Sensor: [Aqua TROLL Temperature Sensor & Conductivity Sensor - In-Situ](https://in-situ.com/us/aqua-troll-temperature-conductivity-sensor?srsltid=AfmBOopE4DwiUreDIF-6juTAGf-mhWxy15_9JYtrftymGbzvsog2NxI-),
2. In-Situ Aqua TROLL™ Temperature-Conductivity data logger (200 model): [Aqua TROLL 200 Data Logger](https://in-situ.com/us/aqua-troll-200-data-logger?srsltid=AfmBOophVpw7ktT1RAdopwrH3LxB6pSXjPAKunaZ9qL-iBD3QFfDRE60), and
3. YSI™ sondes (EXO2 or EXO3 models) equipped with YSI™ EXO wiped conductivity and temperature sensors: [EXO Wiped Conductivity and Temperature Sensor | ysi.com](https://www.ysi.com/wipedct).

Any use of trade, firm, or product names is for descriptive purposes only and does not imply endorsement by the U.S. Government.

**Identifying co-located sites:** Discrete sites were considered co-located with a continuous site if they were located: (1) on the same National Hydrography Dataset (NHD) Plus Version 2.1 (NHDPlusV2.1; 1:100K scale) network (McKay et al., 2012) flowline, and the flowline was less than 1.5 kilometers long, or (2) on the same NHD flowline and the distance between continuous and discrete monitoring sites was no more than 10 percent of the length of the flowline. An additional 10 discrete sites were identified as co-located with continuous SC sites using this approach.

**Discrete SC-chloride paired observations**: We selected the 2010 to 2022 temporal window for discrete data compilation to ensure the SC-chloride regression models were developed with recent data to reflect contemporary conditions, while also maximizing spatial coverage across the region to accurately reflect water-quality conditions for each cluster. The selected dataset contained almost 9,000 discrete measurements of SC and chloride across 156 sites within the region. Data were evenly spread across twelve years (ranging from 445-897 observations per year) except for 2022, with only 31 observations. This was because of lags in data approval and entry (our dataset was compiled in July 2022).

**Screening for tidal influences:** Tidally influenced stations were identified based on the historic location of the salt front near river mile 110 of the Delaware River in Philadelphia, PA as approximated by data from the Delaware River Basin Commission (2025). All sites on the main stem of the Delaware River-- downstream of river mile 110--were omitted from cluster analysis. Three additional sites, near Delaware Bay, were omitted because of high diurnal SC measurements that were likely reflective of tidally influenced seawater inputs. Tidal sites were excluded from cluster analysis because the SC vs chloride relation is controlled by sea water as opposed to deicing salt or other anthropogenic sources.

**Outlier assessment:** Outliers were evaluated using methods from Moore et al. (2020). Discrete measures of chloride and SC were imported into R and analyzed using the base “lm” function (version 4.2.0) to fit a linear model for each site. Then, data values were ranked and subsequently flagged to be screened for removal if the resulting leverage or Cook’s distance fell within the top 5 values, and if the standardized residual of the data value was equal to + or – 4. Data values flagged because of high leverage, high Cook’s distance, or high or low standardized residuals were then manually inspected to be considered for removal. Data values were removed if they were flagged for all three of the metrics of concern.

**Data availability:** All data used for, or generated in, the analyses, including discrete measures chloride and SC observations, watershed characteristics, final SC-chloride model regression equations, and daily and hourly chloride predictions, are available in the associated data release (Hardesty and others, 2025).

**Table SI-1**. Information for the watershed characteristics used in cluster analysis, including general variable category, variable name, variable description, scale at which the data were summarized for the Delaware River Basin (watershed = variable was summarized for the delineated watershed using the monitoring location as the pour point; COMID = the variable was summarized using the area for the associated NHDPlusV2.1 network COMID or reach ID; McKay et al. 2012), the summarized data source (if applicable), and the original source. Only variables that were summarized by COMIDs have both data sources and original sources. LULC= land use/land cover. (see separate .xlsx file)

**Table SI-2.** Continuous specific conductance (SC) and predicted chloride data coverage at 82 non-tidal sites for the winter seasons during the 2020-2022 study period, Delaware River Basin. Winter was defined as December 1 the previous year through March 31 of the current year. Data coverage is expressed as a percentage of total days during the season (n = 122, 121, and 121 for years 2020, 2021, and 2022, respectively). Values less than 80% (cut-off for inclusion in analyses) are shaded black while values at or above 80% are shaded gray.

|  | **Percentage of days with data in winter season** | | |  |  | **Percentage of days with data in winter season** | | |
| --- | --- | --- | --- | --- | --- | --- | --- | --- |
| **Site ID** | **2020** | **2021** | **2022** |  | **siteID** | **2020** | **2021** | **2022** |
| BasherKi | 0 | 100 | 99.2 |  | McDonald | 100 | 78.5 | 100 |
| Beaverda | 100 | 97.5 | 100 |  | MidBrWCC | 33.1 | 95.9 | 100 |
| BiscuitB | 44.6 | 100 | 95.9 |  | MillBroo | 100 | 17.4 | 89.3 |
| Brandyw1 | 100 | 100 | 100 |  | Mongaup1 | 91.7 | 0 | 0 |
| Brandyw2 | 37.2 | 33.1 | 53.7 |  | Mongaup2 | 100 | 100 | 100 |
| Brodhead | 100 | 84.3 | 100 |  | NRancoc1 | 70.2 | 100 | 100 |
| BushKill | 96.7 | 100 | 96.7 |  | NRancoc2 | 50.4 | 100 | 100 |
| CedarCrk | 62.8 | 100 | 100 |  | Neshamin | 100 | 100 | 0 |
| Christin | 0 | 0 | 100 |  | NvSkBrid | 62 | 100 | 93.4 |
| CobbsCr1 | 26.4 | 100 | 2.48 |  | NvSkClar | 54.5 | 100 | 100 |
| CobbsCr2 | 26.4 | 100 | 1.65 |  | NvSkDenn | 100 | 100 | 95 |
| CooperR1 | 28.9 | 99.2 | 100 |  | NvSkECla | 62.8 | 100 | 82.6 |
| CooperR2 | 0 | 0 | 85.1 |  | NvSkGode | 54.5 | 100 | 100 |
| CrssWck1 | 100 | 100 | 100 |  | NvSkNeve | 87.6 | 100 | 100 |
| CrssWck2 | 49.6 | 99.2 | 100 |  | NvSkWCla | 66.9 | 99.2 | 93.4 |
| DRBarryv | 100 | 80.2 | 95 |  | NvSkWinn | 92.6 | 100 | 100 |
| DRBelvid | 100 | 100 | 100 |  | Perkiome | 98.3 | 99.2 | 97.5 |
| DRCallic | 92.6 | 94.2 | 89.3 |  | PikeCree | 100 | 100 | 99.2 |
| DRFrench | 98.3 | 95.9 | 95.9 |  | Raccoon1 | 51.2 | 100 | 100 |
| DRLordvi | 94.2 | 100 | 100 |  | Raccoon2 | 0 | 100 | 54.5 |
| DRMargar | 94.2 | 100 | 100 |  | Rancocas | 100 | 95.9 | 99.2 |
| DRMontag | 100 | 96.7 | 90.9 |  | RedClayC | 0 | 0 | 95.9 |
| DRPennyp | 100 | 100 | 70.2 |  | SRancoca | 100 | 100 | 99.2 |
| DRPhilly | 100 | 2.48 | 0 |  | SWRancoc | 16.5 | 100 | 0 |
| DRPortJe | 100 | 79.3 | 95.9 |  | Schuylk1 | 100 | 100 | 100 |
| DRTrento | 95.9 | 100 | 97.5 |  | Schuylk2 | 33.9 | 29.8 | 0 |
| DRWalton | 100 | 37.2 | 92.6 |  | Schuylk3 | 100 | 100 | 0 |
| DarbyCrk | 100 | 100 | 100 |  | Sheldrak | 100 | 98.3 | 100 |
| EBBrandy | 36.4 | 47.1 | 0.826 |  | SilverLk | 0 | 0 | 100 |
| EastWCC1 | 62.8 | 100 | 100 |  | Spennsau | 100 | 72.7 | 0 |
| EastWCC2 | 100 | 0 | 0 |  | SpringCr | 17.4 | 100 | 100 |
| FlatBroo | 47.1 | 100 | 100 |  | StJonesR | 0 | 0 | 100 |
| Frankfor | 100 | 96.7 | 29.8 |  | TremperK | 100 | 83.5 | 98.3 |
| Greenwoo | 100 | 100 | 95.9 |  | TroutRun | 97.5 | 100 | 100 |
| GumaerBk | 90.9 | 100 | 100 |  | ValleyCr | 100 | 100 | 100 |
| JordanCr | 100 | 100 | 100 |  | WBBrandy | 100 | 100 | 100 |
| LLehigh2 | 99.2 | 100 | 100 |  | WCCNewr1 | 69.4 | 100 | 98.3 |
| LLehigh3 | 88.4 | 100 | 100 |  | WCCNewr2 | 100 | 100 | 100 |
| LehighGl | 5.79 | 0 | 4.96 |  | WCCStant | 0 | 0 | 100 |
| LeibertC | 0 | 100 | 99.2 |  | WCCStric | 100 | 93.4 | 100 |
| Llehigh1 | 100 | 100 | 100 |  | WstBrWCC | 21.5 | 100 | 100 |

**Table SI-3.** Variable loadings for top five principal components (PC) from principal component analysis, Delaware River Basin. See Table SI-1 for variable definitions. LULC = land use/land cover. Gray shading indicates the five watershed characteristics with the highest loadings associated with each PC.

| **Variable category** | **Variable name** | **Principal component** | **Variable loading** | **Absolute value of variable loading** |
| --- | --- | --- | --- | --- |
| LULC | lc_forest_pct | PC1 | 0.25 | 0.25 |
| LULC | lc_devel_pct | PC1 | -0.24 | 0.24 |
| surficial lithology | sl_nonglacial | PC1 | -0.24 | 0.24 |
| surficial lithology | sl_glacial | PC1 | 0.24 | 0.24 |
| major lithology | ml_sedapp_pct | PC1 | 0.24 | 0.24 |
| major lithology | ml_uncon_pct | PC2 | -0.30 | 0.30 |
| LULC | lc_wetla_pct | PC2 | -0.30 | 0.30 |
| LULC | lc_wetla_pct_Cat | PC2 | -0.24 | 0.24 |
| lithologic geochemistry | CaOWs | PC2 | 0.24 | 0.24 |
| lithologic geochemistry | MgOWs | PC2 | 0.24 | 0.24 |
| lithologic geochemistry | SiO2Ws | PC3 | 0.35 | 0.35 |
| lithologic geochemistry | CaOWs | PC3 | -0.35 | 0.35 |
| major lithology | ml_carbo_pct | PC3 | -0.34 | 0.34 |
| lithologic geochemistry | MgOWs | PC3 | -0.32 | 0.32 |
| surficial lithology | PctCarbResidWs | PC3 | -0.30 | 0.30 |
| mining | ACC_NWALT12_41 | PC4 | -0.39 | 0.39 |
| LULC | lc_barren_pct | PC4 | -0.33 | 0.33 |
| LULC | lc_water_pct_Cat | PC4 | -0.32 | 0.32 |
| basin area | area_km2 | PC4 | -0.31 | 0.31 |
| LULC | lc_herba_pct | PC4 | -0.30 | 0.30 |
| contaminant point source density | MineDensWs | PC5 | -0.53 | 0.53 |
| contaminant point source density | MineDensCat | PC5 | -0.52 | 0.52 |
| contaminant point source density | MineDensWsRp100 | PC5 | -0.30 | 0.30 |
| LULC | lc_agric_pct | PC5 | 0.23 | 0.23 |
| contaminant point source density | SuperfundDensWs | PC5 | -0.18 | 0.18 |

**Table SI-4.** Characteristics of three winter seasons for the three-year study period (2020-2022) for two cities within the Delaware River Basin. Number of events are the number of days during the season where snowfall was more than or equal to 0.1 inch (measurable snow). The Accumulated Winter Season Severity Index is a composite index provided by the Midwest Regional Climate Center (2025) to describe relative severity of winter seasons (Boustead et al., 2015).

| **City** | **Season** | **Start** | **End** | **Number of events** | **Accumulated Winter Season Severity Index** |
| --- | --- | --- | --- | --- | --- |
| Allentown, PA | 2019-2020 | 12/1/2019 | 2/29/2020 | 6 | 124 |
|  | 2020-2021 | 12/1/2020 | 3/2/2021 | 17 | 469 |
|  | 2021-2022 | 12/1/2021 | 3/28/2022 | 12 | 313 |
| Philadelphia, PA | 2019-2020 | 12/1/2019 | 2/29/2020 | 2 | 70 |
|  | 2020-2021 | 12/1/2020 | 2/28/2021 | 12 | 166 |
|  | 2021-2022 | 12/1/2021 | 3/12/2022 | 7 | 142 |

**Table SI-5.** The number of exceedance events, the total duration of exceedance events, and the median duration of exceedance events above the Environmental Protection Agency (EPA) chronic criterion (230 mg L^-1^) for the period 2020-2022 computed using predicted daily mean concentrations derived from the 5^th^, 50^th^, and 95^th^ percentile prediction interval sub-daily chloride concentration values (denoted as “lower”, “median” and “upper”, respectively), Delaware River Basin.

|  |  | **Number of chronic events** | | |  | **Total duration, days** | | |  | **Median event duration, days** | | |
| --- | --- | --- | --- | --- | --- | --- | --- | --- | --- | --- | --- | --- |
| **site ID** |  | **Lower** | **Median** | **Upper** |  | **Lower** | **Median** | **Upper** |  | **Lower** | **Median** | **Upper** |
| CedarCrk |  | 3 | 4 | 4 |  | 9 | 10 | 13 |  | 3 | 2.5 | 3 |
| Christin |  | NA | NA | 1 |  | NA | NA | 1 |  | NA | NA | 1 |
| CobbsCr1 |  | 3 | 2 | 2 |  | 30 | 32 | 34 |  | 9 | 16 | 17 |
| CobbsCr2 |  | 2 | 2 | 2 |  | 33 | 35 | 39 |  | 16.5 | 17.5 | 19.5 |
| CooperR1 |  | 5 | 5 | 6 |  | 52 | 55 | 59 |  | 8 | 8 | 7.5 |
| DarbyCrk |  | 8 | 8 | 8 |  | 70 | 75 | 82 |  | 6 | 7 | 8 |
| Frankfor |  | 5 | 4 | 7 |  | 40 | 43 | 52 |  | 7 | 7 | 3 |
| Neshamin |  | 2 | 2 | 2 |  | 7 | 10 | 14 |  | 3.5 | 5 | 7 |
| Perkiome |  | NA | NA | 1 |  | NA | NA | 1 |  | NA | NA | 1 |
| PikeCree |  | 1 | 1 | 3 |  | 3 | 3 | 11 |  | 3 | 3 | 4 |
| Spennsau |  | 3 | 3 | 3 |  | 38 | 40 | 42 |  | 9 | 10 | 10 |
| SpringCr |  | NA | NA | 2 |  | NA | NA | 2 |  | NA | NA | 1 |
| TroutRun |  | NA | NA | 5 |  | NA | NA | 14 |  | NA | NA | 3 |
| ValleyCr |  | 5 | 5 | 9 |  | 27 | 39 | 56 |  | 4 | 4 | 4 |
| WCCNewr2 |  | NA | NA | 2 |  | NA | NA | 2 |  | NA | NA | 1 |

**Table SI-6.** The number of exceedance events, the total duration of exceedance events, and the median duration of exceedance events above the Environmental Protection Agency acute criterion (860 mg L^-1^) for the period 2020-2022 computed using predicted hourly mean concentrations derived from the 5^th^, 50^th^, and 95^th^ percentile prediction interval sub-hourly chloride concentration values (denoted as “lower”, “median” and “upper”, respectively), Delaware River Basin.

|  |  | **Number of acute events** | | |  | **Total duration, hours** | | |  | **Median event duration, hours** | | |
| --- | --- | --- | --- | --- | --- | --- | --- | --- | --- | --- | --- | --- |
| **Site ID** |  | **Lower** | **Median** | **Upper** |  | **Lower** | **Median** | **Upper** |  | **Lower** | **Median** | **Upper** |
| CedarCrk |  | 1 | 1 | 2 |  | 1 | 3 | 8 |  | 1 | 3 | 4 |
| CobbsCr1 |  | 6 | 6 | 6 |  | 152 | 162 | 169 |  | 23.5 | 26 | 26.5 |
| CobbsCr2 |  | 9 | 9 | 8 |  | 308 | 326 | 352 |  | 52 | 54 | 57.5 |
| CooperR1 |  | 9 | 9 | 9 |  | 218 | 230 | 241 |  | 24 | 27 | 28 |
| DarbyCrk |  | 7 | 8 | 9 |  | 53 | 61 | 67 |  | 4 | 6 | 6 |
| Frankfor |  | 6 | 6 | 6 |  | 136 | 152 | 167 |  | 20 | 22.5 | 24.5 |
| Spennsau |  | 11 | 11 | 11 |  | 458 | 466 | 476 |  | 29 | 30 | 31 |
| TroutRun |  | NA | NA | 1 |  | NA | NA | 1 |  | NA | NA | 1 |
| ValleyCr |  | NA | 2 | 3 |  | NA | 8 | 22 |  | NA | 4 | 7 |

**Table SI-7.** The number of exceedance events, the total duration of exceedance events, and the median duration of exceedance events above the Canadian Water Quality Guideline chronic criterion (120 mg L^-1^) for the period 2020-2022 computed using predicted daily mean concentrations derived from the 5^th^, 50^th^, and 95^th^ percentile prediction interval sub-daily chloride concentration values (denoted as “lower”, “median” and “upper”, respectively), Delaware River Basin.

|  |  | **Number of chronic events** | | |  | **Total duration, days** | | |  | **Median event duration, days** | | |
| --- | --- | --- | --- | --- | --- | --- | --- | --- | --- | --- | --- | --- |
| **Site ID** |  | **Lower** | **Median** | **Upper** |  | **Lower** | **Median** | **Upper** |  | **Lower** | **Median** | **Upper** |
| Brandyw1 |  | 3 | 5 | 4 |  | 5 | 8 | 12 |  | 2 | 2 | 2.5 |
| CedarCrk |  | 18 | 17 | 26 |  | 62 | 83 | 131 |  | 2 | 3 | 3 |
| Christin |  | 2 | 5 | 5 |  | 10 | 15 | 19 |  | 5 | 3 | 4 |
| CobbsCr1 |  | 3 | 35 | 62 |  | 35 | 184 | 600 |  | 7 | 3 | 7 |
| CobbsCr2 |  | 3 | 66 | 63 |  | 39 | 422 | 579 |  | 8 | 4 | 7 |
| CooperR1 |  | 7 | 8 | 8 |  | 96 | 112 | 135 |  | 11 | 13.5 | 5.5 |
| CooperR2 |  | NA | 1 | 2 |  | NA | 1 | 5 |  | NA | 1 | 2.5 |
| DarbyCrk |  | 45 | 75 | 95 |  | 265 | 508 | 729 |  | 2 | 4 | 5 |
| Frankfor |  | 52 | 77 | 78 |  | 277 | 484 | 602 |  | 3 | 4 | 5.5 |
| LLehigh2 |  | 2 | 3 | 4 |  | 3 | 5 | 6 |  | 1.5 | 2 | 1.5 |
| LLehigh3 |  | 5 | 6 | 10 |  | 8 | 14 | 26 |  | 2 | 2 | 2 |
| LeibertC |  | NA | NA | 6 |  | NA | NA | 6 |  | NA | NA | 1 |
| Neshamin |  | 3 | 6 | 24 |  | 24 | 35 | 133 |  | 8 | 2 | 2 |
| Perkiome |  | 2 | 2 | 5 |  | 15 | 18 | 28 |  | 7.5 | 9 | 2 |
| PikeCree |  | 8 | 11 | 10 |  | 12 | 24 | 47 |  | 1.5 | 2 | 4 |
| Raccoon2 |  | NA | NA | 1 |  | NA | NA | 1 |  | NA | NA | 1 |
| RedClayC |  | NA | NA | 2 |  | NA | NA | 2 |  | NA | NA | 1 |
| SWRancoc |  | NA | 2 | 4 |  | NA | 2 | 9 |  | NA | 1 | 2 |
| Schuylk1 |  | 1 | 1 | 2 |  | 1 | 1 | 2 |  | 1 | 1 | 1 |
| Schuylk2 |  | NA | NA | 1 |  | NA | NA | 1 |  | NA | NA | 1 |
| Schuylk3 |  | NA | NA | 3 |  | NA | NA | 12 |  | NA | NA | 4 |
| Sheldrak |  | 3 | 2 | 2 |  | 3 | 7 | 8 |  | 1 | 3.5 | 4 |
| Spennsau |  | 7 | 13 | 40 |  | 49 | 99 | 333 |  | 3 | 4 | 6 |
| SpringCr |  | 7 | 11 | 16 |  | 15 | 21 | 33 |  | 2 | 1 | 1.5 |
| TroutRun |  | 5 | 13 | 63 |  | 9 | 46 | 900 |  | 2 | 2 | 9 |
| ValleyCr |  | 73 | 76 | 67 |  | 623 | 735 | 788 |  | 6 | 5 | 6 |
| WCCNewr1 |  | NA | NA | 1 |  | NA | NA | 1 |  | NA | NA | 1 |
| WCCNewr2 |  | 5 | 9 | 11 |  | 15 | 21 | 27 |  | 2 | 2 | 2 |
| WCCStant |  | 1 | 4 | 5 |  | 1 | 4 | 6 |  | 1 | 1 | 1 |

**Table SI-8.** Winter 2021 snow depth, length of roads serviced, and amount of deicer material applied to highways in a county for some study sites, Delaware River Basin (Pennsylvania Department of Transportation 2021).

| **Site ID** | **Carbonate in watershed** | **District** | **County** | **Length of snow lanes serviced, km** | **Snow, mm** | **Total road salt application, kg** | **Total brine applied, liters** |
| --- | --- | --- | --- | --- | --- | --- | --- |
| ValleyCr | Yes | 6 | Chester | 3,182 | 56 | 10,770,000 | 73,000 |
| TroutRun | No |  |  |  |  |  |  |
| SpringCr | Yes | 5 | Lehigh | 2,295 | 56 | 8,880,000 | 1,071,000 |
| LLehigh3 | Yes |  |  |  |  |  |  |
| CedarCrk | Yes |  |  |  |  |  |  |
| LLehigh2 | Yes |  |  |  |  |  |  |
| Neshamin | No | 6 | Bucks | 5,169 | 41 | 15,310,000 | 191,000 |
| Frankfor | No | 6 | Philadelphia | 2,216 | 25 | 15,000,000 | 649,000 |
| CobbsCr1 | No |  |  |  |  |  |  |
| DarbyCrk | No | 6 | Delaware | 2,628 | 18 | 22,370,000 | 225,000 |
| CobbsCr2 | No |  |  |  |  |  |  |

*
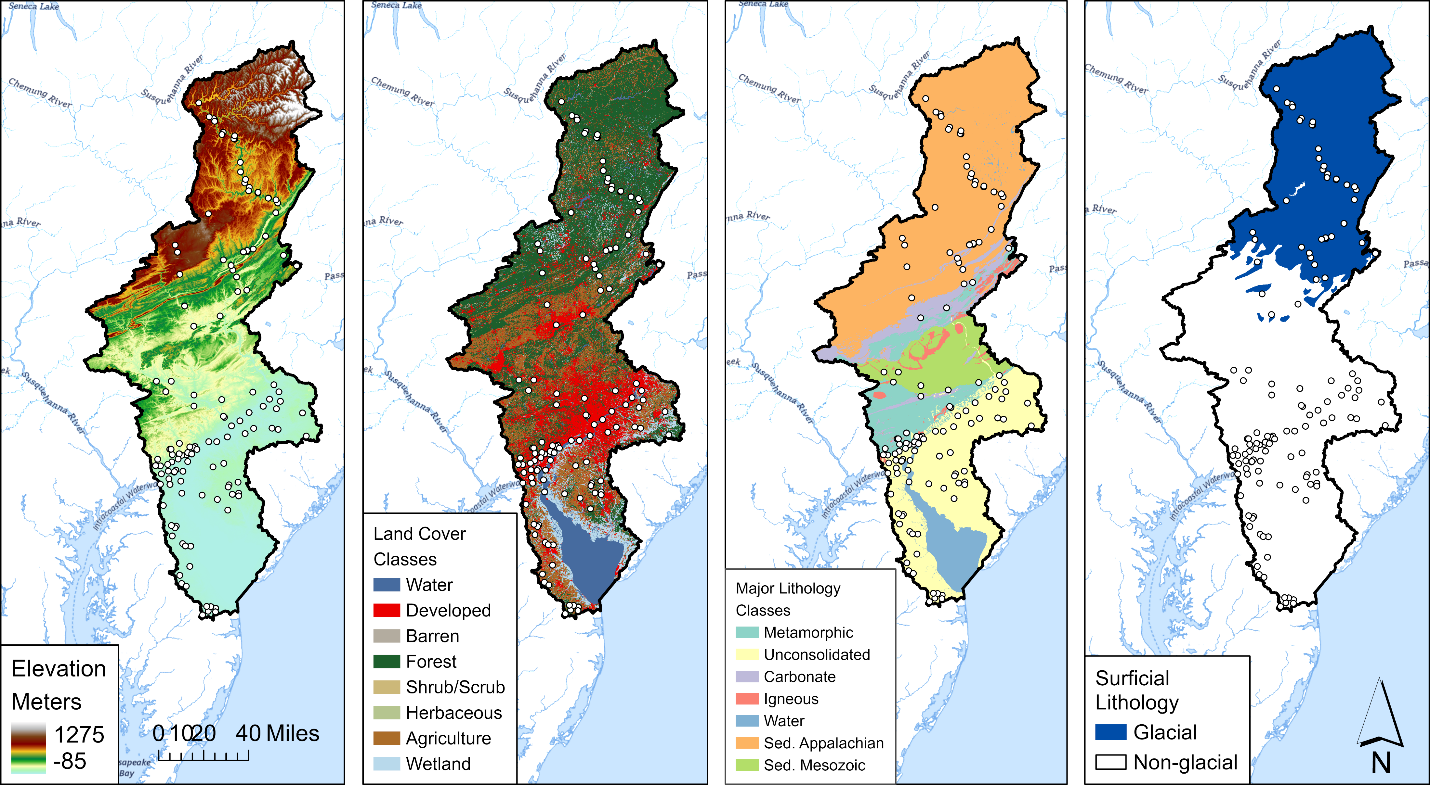
*

**Fig. SI-1.** Maps showing spatial distribution of watershed characteristics of the Delaware River Basin: (A) elevation, (B) land use/land cover, (C) major lithology, and (D) surficial lithology. Dots indicate location of the 156 discrete water quality sites. Base map uses data from the U.S. Geological Survey, The National Map, 2023.


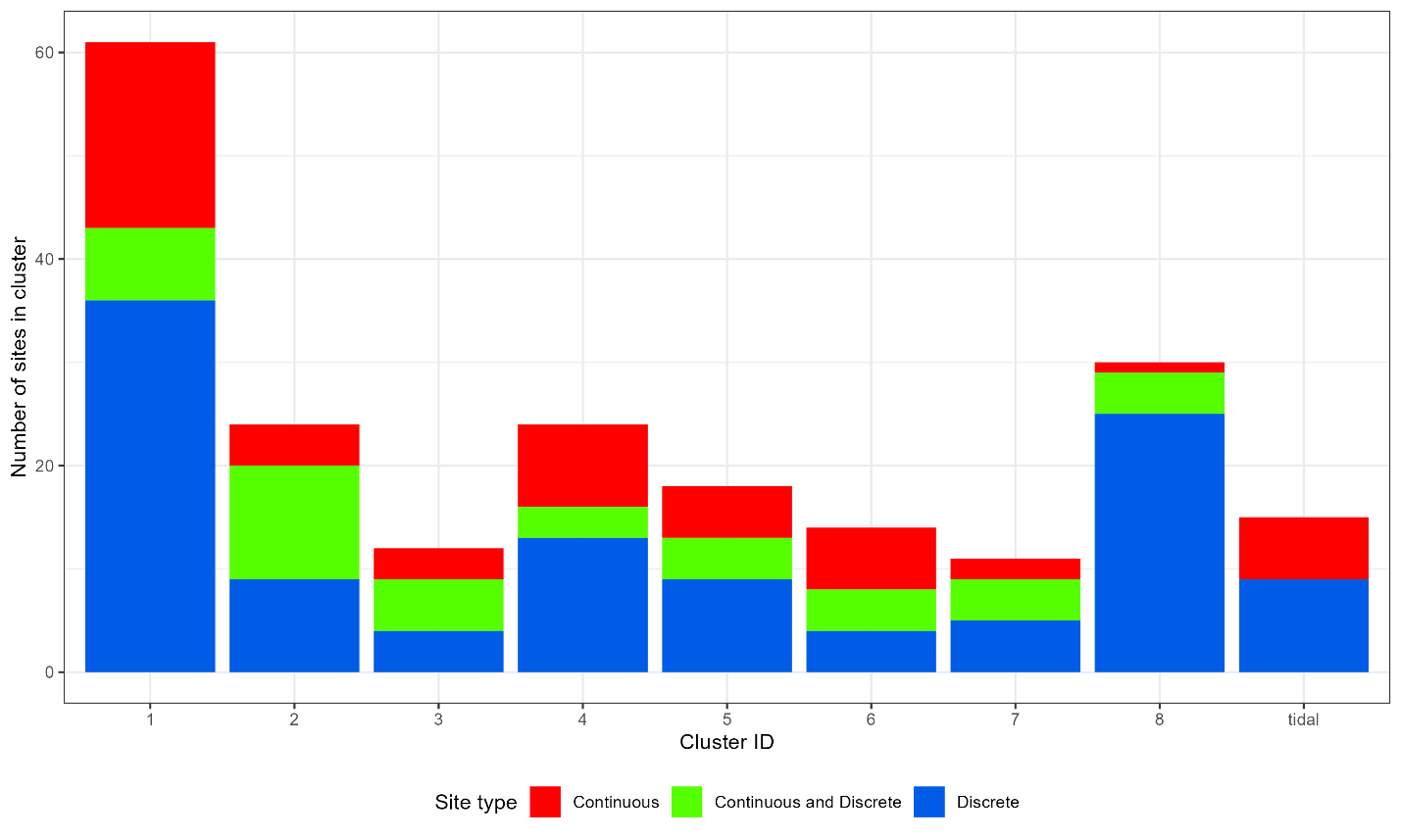
**Fig. SI-2**. Bar plot showing number of sites contained in each cluster and count of site types among the nine clusters for this study in the Delaware River Basin.


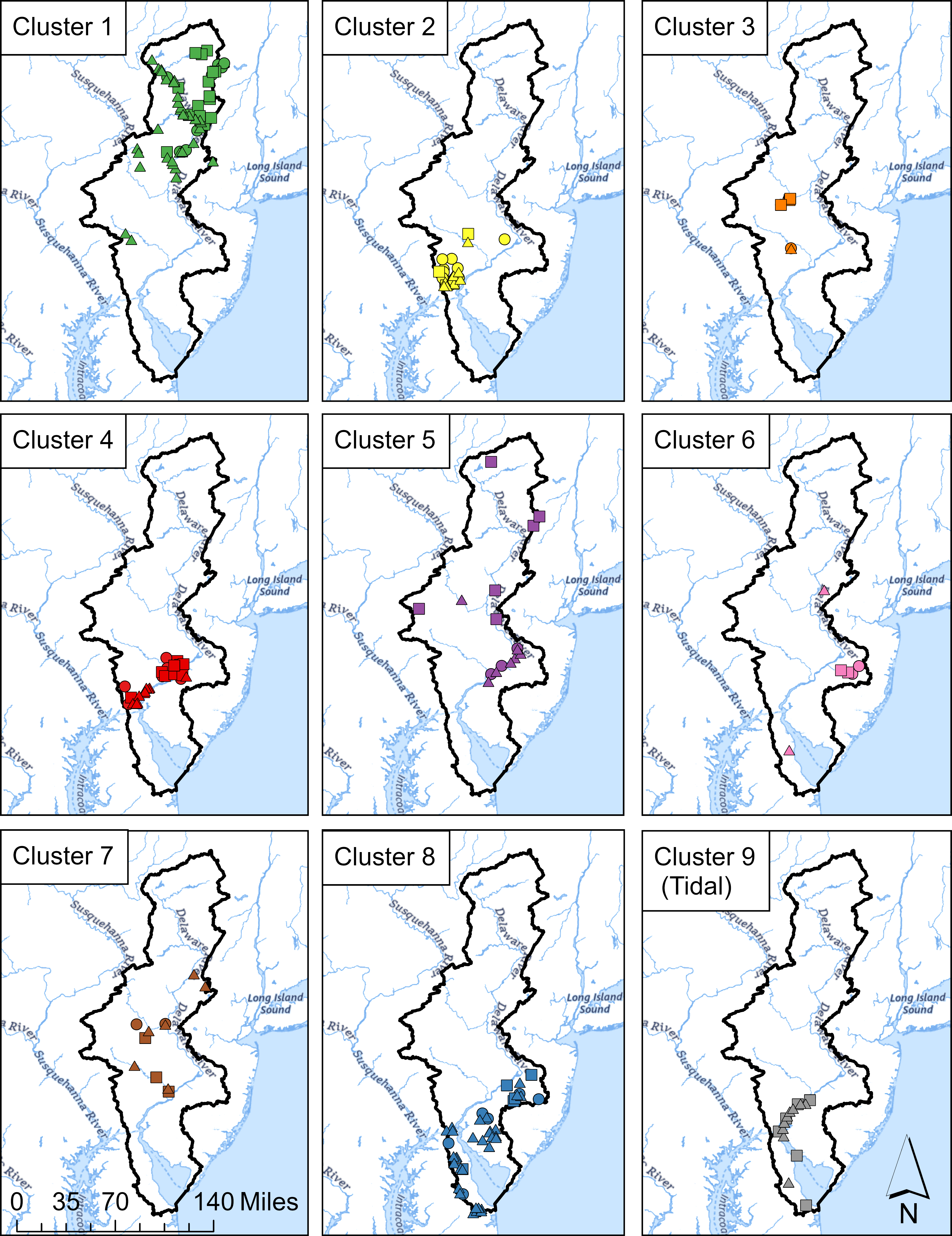


**Fig. SI-3.** Maps showing spatial distribution of each of the nine clusters across the Delaware River Basin. Circles represent sites with continuous and discrete data; squares represent sites with only continuous data; triangles represent sites with only discrete data. Base map uses data from the U.S. Geological Survey, The National Map, 2023.


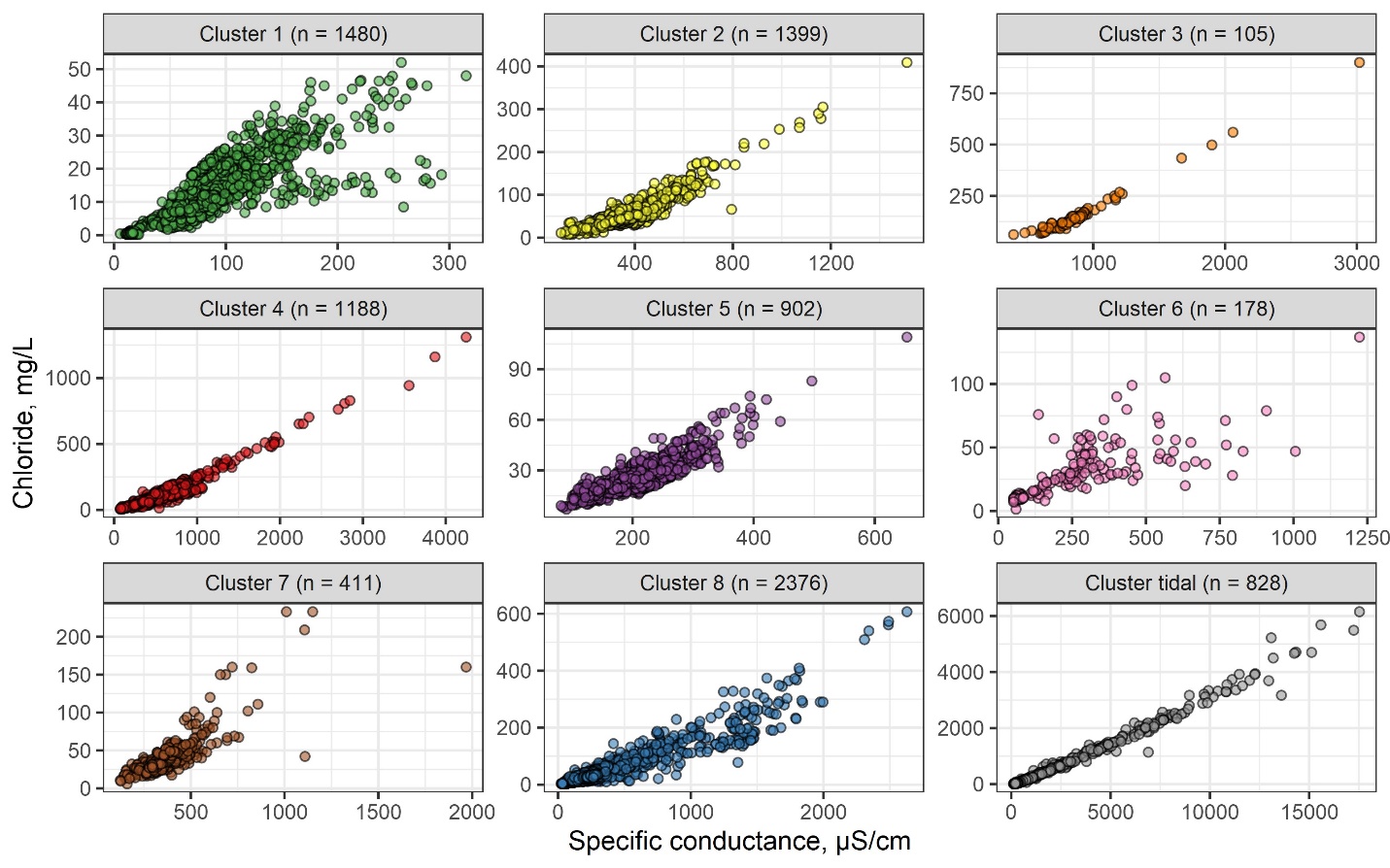


**Fig. SI-4**. Scatter plot of discrete measures of specific conductance (x-axis) and chloride (y-axis) at all sites with discrete data for the nine clusters in the Delaware River Basin. Note that axis limits differ between panels. n = number of observations in each plot/cluster.


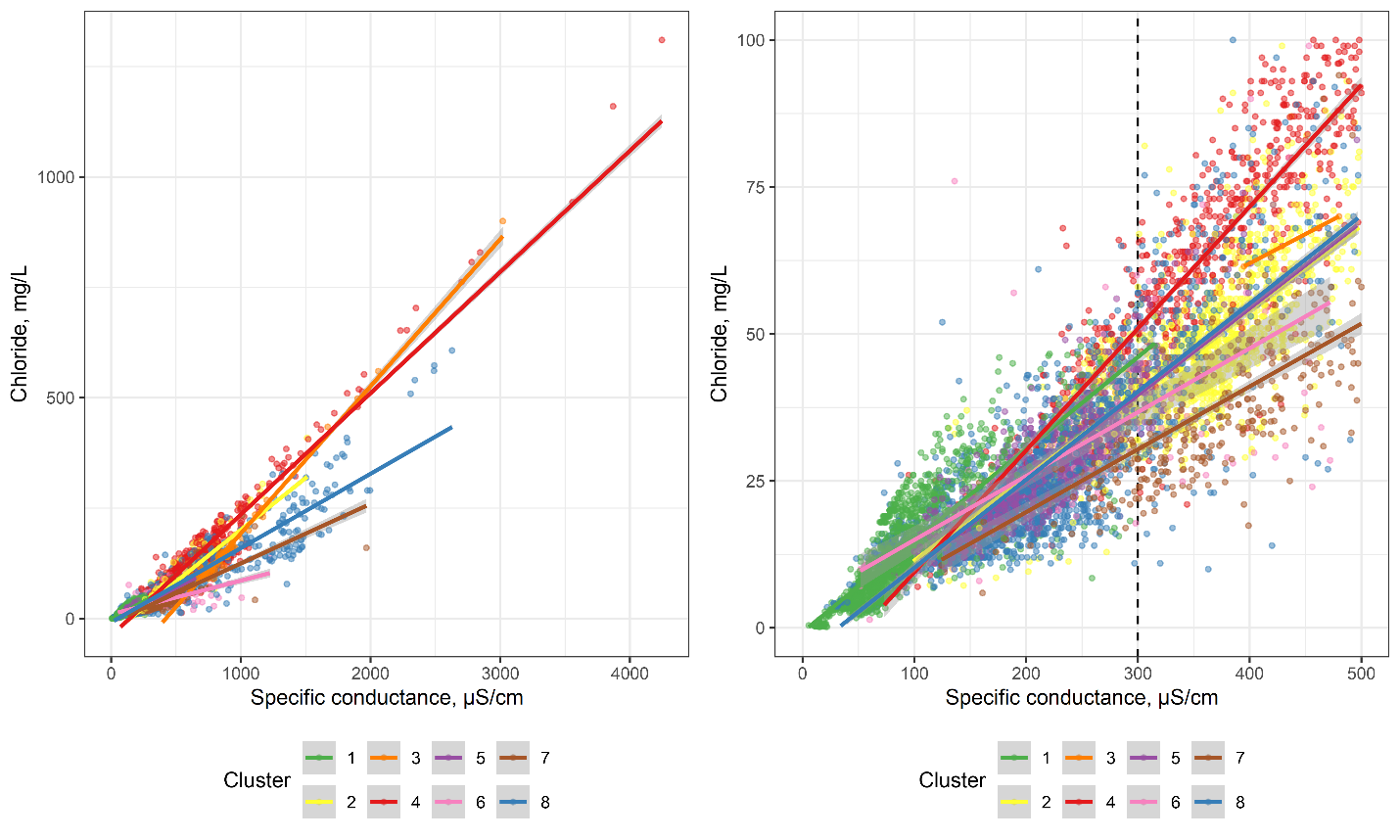


**Fig. SI-5**. Scatter plots and linear relations between measures of specific conductance (SC) and chloride concentration. Left panel shows relationship between chloride and specific conductance (SC) measurements for the eight non-tidal clusters in the Delaware River Basin. Right panel shows the same plot as the left but with axis limits reduced (chloride < 100 mg L^-1^ and SC < 500 µS cm^-1^) for the eight non-tidal clusters. Colored lines denote the general relation between specific conductance and chloride using the *geom_smooth* function (method='lm', formula= y~x; Wickham, 2016) for data contained within the plot and do not reflect the final regression models used for prediction; these lines are used only to visualize the relation between SC and chloride. Dashed vertical line on the right panel denotes 300 µS cm^-1^for specific conductance.


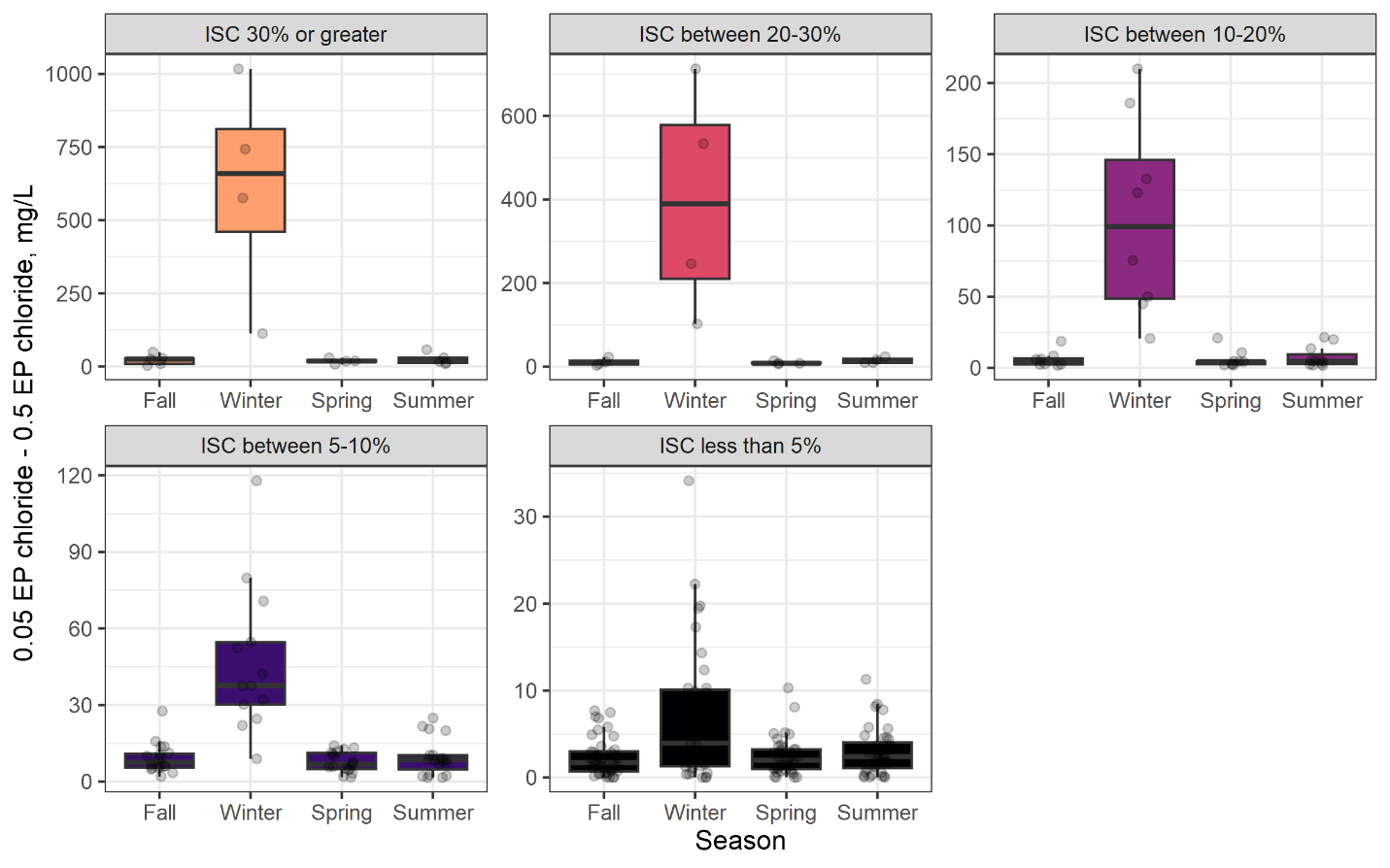


**Fig. SI-6**. Box plots showing the difference between 0.05 exceedance probability (EP) predicted chloride concentrations and 0.5 exceedance probability predicted chloride concentrations for the four seasons for water year 2021 across the five impervious surface cover (ISC) categories shown in Figure 4b, Delaware River Basin.


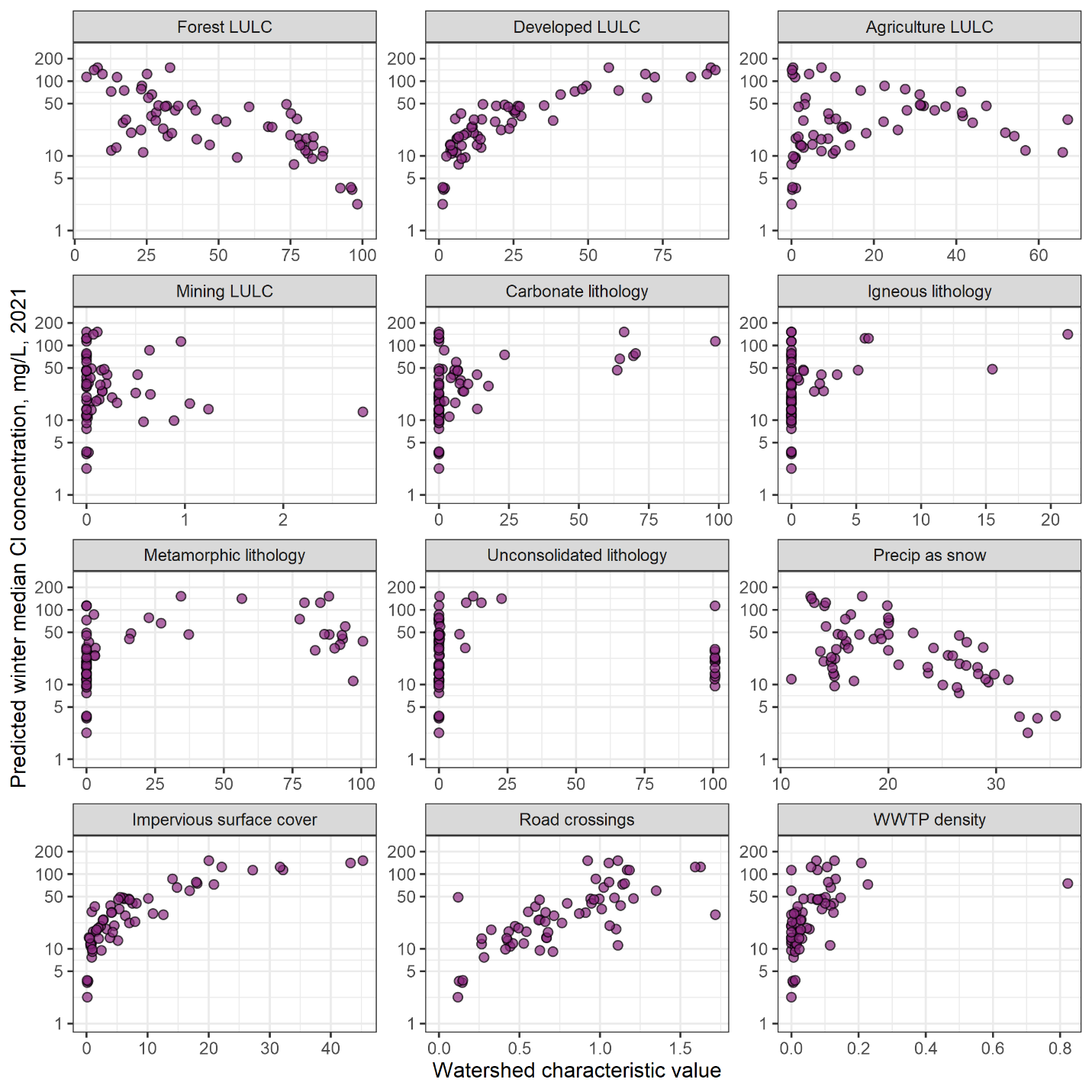


**Fig. SI-7**. Scatter plots of predicted median (0.5 exceedance probability) winter chloride concentrations calculated from predicted daily mean chloride concentrations for water year 2021 versus watershed characteristics, including: percentage land use/land cover (LULC; forest, developed, agriculture, mining); percentage major lithology (carbonate, igneous, metamorphic, and unconsolidated); percentage of annual precipitation as snow; and several additional urban development variables, including percentage impervious cover, number of road crossings (count per km^2^), and wastewater treatment plant (WWTP) density (facilities per km^2^), Delaware River Basin.

**
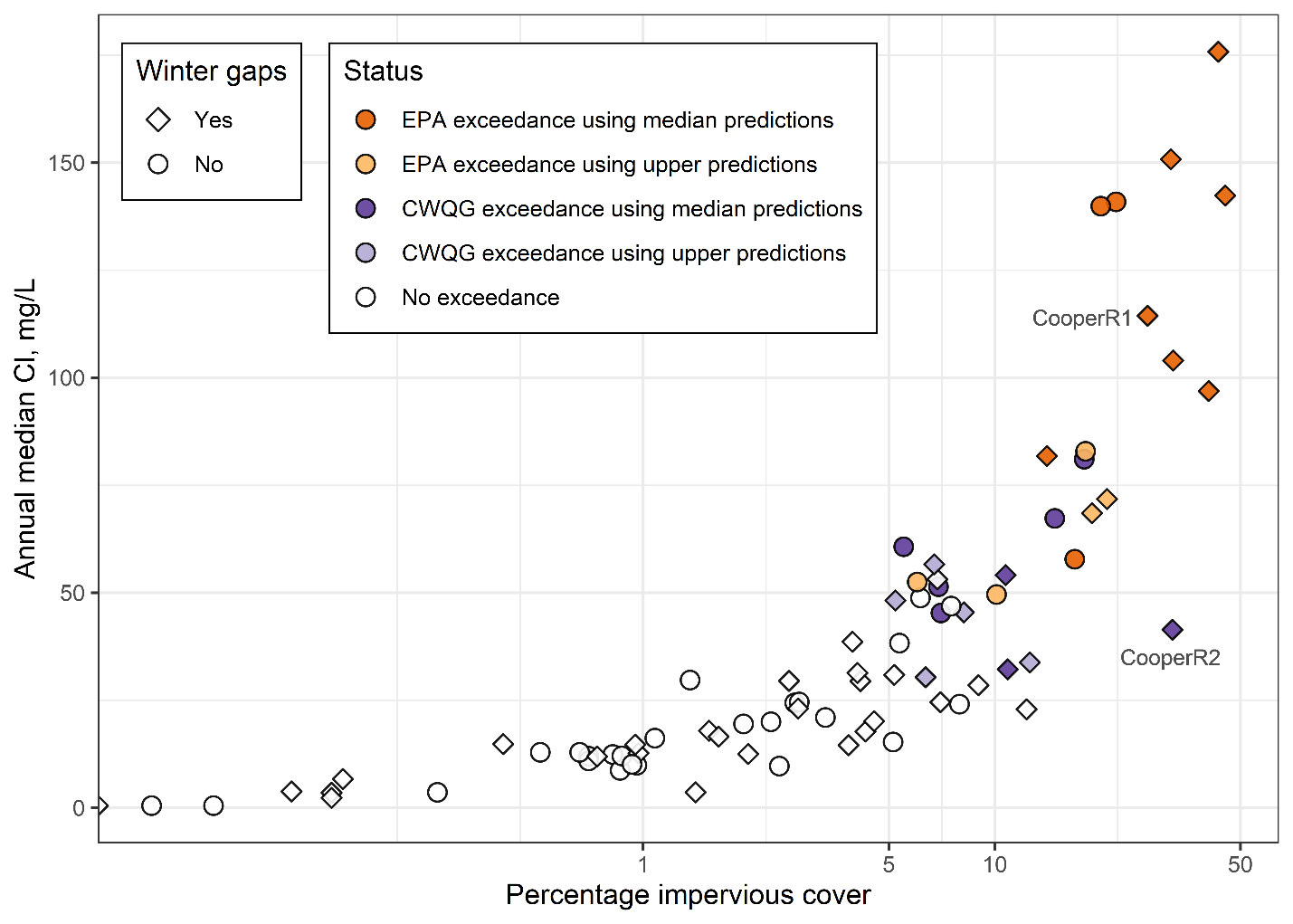
**

**Fig. SI-8**: Alternative version of Figure 8, shown with median annual chloride. Percentage impervious surface cover versus predicted annual median chloride (Cl) concentrations calculated from predicted daily mean chloride concentrations for 2020-2022 for the 82 non-tidal continuous SC monitoring sites in the Delaware River Basin. Sites where chloride exceeded the U.S. Environmental Protection Agency (EPA) water quality criteria or Canadian Water Quality Guidelines (CWQG) chronic criteria are denoted by their color. Diamond shapes represent sites with a substantial data gap during the winter season (greater than 20% of the season with missing data) for at least one of the three winter seasons.

**
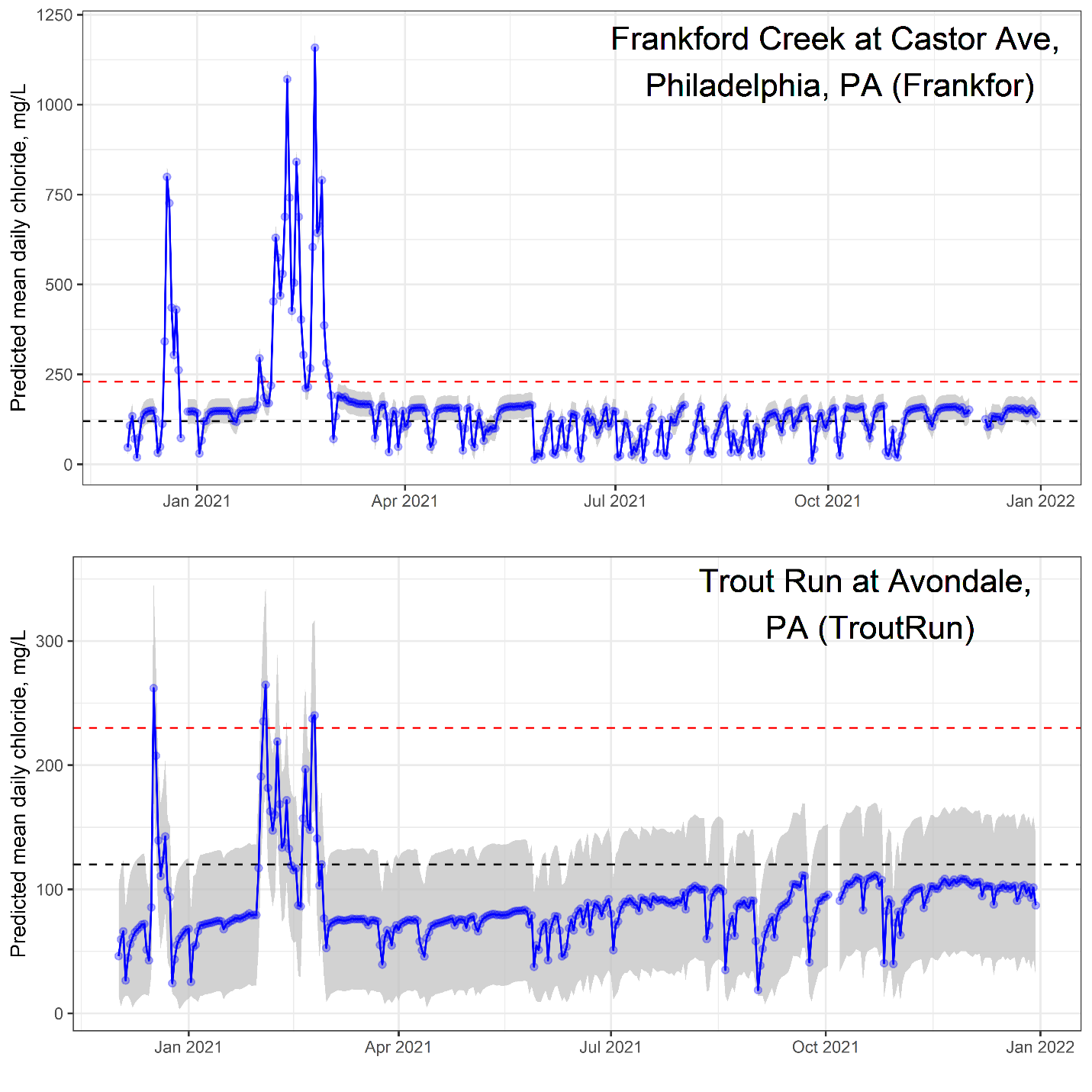
**

**Fig. SI-9**. Predicted daily mean chloride, mg L^-1^, for December 2020 through December 2021 for two sites within the continuous specific conductance (SC) network in the Delaware River Basin. Blue lines and dots indicate median (50^th^ percentile prediction interval) predicted chloride concentrations. Gray bands denote the 5^th^ and 95^th^ percentile predictions (*i.e.*, lower and upper chloride predictions). Black dashed line indicates the chronic Canada Water Quality Guideline for chloride (CWQG; 120 mg L^-1^). Red dashed line indicates the Environmental Protection Agency chronic threshold (230 mg L^-1^).

**References**

Boustead, B. E. M., S. D. Hilberg, M. D. Shulski, and K.h G. Hubbard. The Accumulated Winter Season Severity Index (AWSSI). Journal of Applied Meteorology and Climatology, Vol. 54, No. 8, August 2015: 1693-1712.DOI: <https://doi.org/10.1175/JAMC-D-14-0217.1>.

Delaware River Basin Commission. 2025. Location of the Delaware River Salt Front website. URL: <https://nj.gov/drbc/programs/flow/salt-front.html#1>. (Accessed July 24 2025).

Dewitz, J., U.S. Geological Survey, 2021. National Land Cover Database (NLCD) 2019 Products (ver. 3.0, February 2024): U.S. Geological Survey data release. <https://doi.org/10.5066/P9KZCM54>.

Hardesty, D. M., R. M. Fanelli, and M. Morency. 2025. Discrete chloride and specific conductance (SC) observations, chloride-SC regression equations, and daily and hourly SC values and chloride predictions for 88 USGS water quality monitoring stations in the Delaware River Basin, 2019-2022. U.S. Geological Survey Data Release. <https://doi.org/10.5066/P146TC5X>.

Hill, R.A., Weber, M.H., Leibowitz, S.G., Olsen, A.R., Thornbrugh, D.J., 2016. The Stream-Catchment (StreamCat) Dataset: A Database of Watershed Metrics for the Conterminous United States. JAWRA Journal of the American Water Resources Association 52, 120–128. <https://doi.org/10.1111/1752-1688.12372>.

Horton, J., 2017. The State Geologic Map Compilation (SGMC) geodatabase of the conterminous United States (ver. 1.1, August 2017): U.S. Geological Survey data release, <https://doi.org/10.5066/F7WH2N65>.

McKay, L., Bondelid, T., Johnston, J., Moore, R., Rhea, A., 2012. NHDPlus Version 2: User Guide.

Midwest Regional Climate Center. 2025. Accumulated Winter Weather Severity Index. <https://mrcc.purdue.edu/research/awssi>. (Data retrieved June 25, 2025).

Moore, J., Fanelli, R.M., Sekellick, A.J., 2020. High-Frequency Data Reveal Deicing Salts Drive Elevated Specific Conductance and Chloride along with Pervasive and Frequent Exceedances of the U.S. Environmental Protection Agency Aquatic Life Criteria for Chloride in Urban Streams. Environ. Sci. Technol. 54, 778–789. <https://doi.org/10.1021/acs.est.9b04316>

Pennsylvania Department of Transportation 2021. PA DOT 2021 Winter Services Guide. PUB 628 (10-21). <https://www.pa.gov/content/dam/copapwp-pagov/en/penndot/documents/public/pubsforms/publications/pub%20628.pdf> (Accessed 7/23/2025).

Wieczorek, M.E., Jackson, S.E., Schwartz, G.E., 2018. Select Attributes for NHDPlus Version 2.1 Reach Catchments and Modified Network Routed Upstream Watersheds for the Conterminous United States. <https://doi.org/10.5066/F7765D7V>.

Wickham, H. 2016. ggplot2: Elegant Graphics for Data Analysis. Springer-Verlag New York. ISBN: 978-3-319-24277-4. URL: <https://ggplot2.tidyverse.org> (Accessed 7/24/2025).
